# Recent Advances in Spectrally Selective Daytime Radiative Cooling Materials

**DOI:** 10.1007/s40820-025-01771-8

**Published:** 2025-05-20

**Authors:** An-Quan Xie, Hui Qiu, Wangkai Jiang, Yu Wang, Shichao Niu, Ke-Qin Zhang, Ghim Wei Ho, Xiao-Qiao Wang

**Affiliations:** 1 National Engineering Laboratory for Modern Silk, College of Textile and Clothing Engineering, Soochow University, Suzhou, 215123 People’s Republic of China; 2 Key Laboratory of Bionic Engineering, Ministry of Education, Jilin University, Changchun, 130022 People’s Republic of China; 3 Institute of Structured and Architected Materials, Liaoning Academy of Materials, Shenyang, 110167 People’s Republic of China; 4 Department of Electrical and Computer Engineering, National University of Singapore, 4 Engineering Drive 3, Singapore, 117583 Singapore

**Keywords:** Daytime radiative cooling, Spectrum-selective emission, Metamaterials, Personal thermal management, Energy harvesting

## Abstract

Daytime radiative cooling is an eco-friendly and passive cooling technology that operates without external energy input. Materials designed for this purpose are engineered to possess high reflectivity in the solar spectrum and high emissivity within the atmospheric transmission window. Unlike broadband-emissive daytime radiative cooling materials, spectrally selective daytime radiative cooling (SSDRC) materials exhibit predominant mid-infrared emission in the atmospheric transmission window. This selective mid-infrared emission suppresses thermal radiation absorption beyond the atmospheric transmission window range, thereby improving the net cooling power of daytime radiative cooling. This review elucidates the fundamental characteristics of SSDRC materials, including their molecular structures, micro- and nanostructures, optical properties, and thermodynamic principles. It also provides a comprehensive overview of the design and fabrication of SSDRC materials in three typical forms, i.e., fibrous materials, membranes, and particle coatings, highlighting their respective cooling mechanisms and performance. Furthermore, the practical applications of SSDRC in personal thermal management, outdoor building cooling, and energy harvesting are summarized. Finally, the challenges and prospects are discussed to guide researchers in advancing SSDRC materials.

## Highlights

- This review comprehensively presents recent advancements in spectrally selective daytime radiative cooling (SSDRC) materials, focusing on their fundamental characteristics, primarily concerning their structures and properties.
- The fabrication principles and corresponding operational mechanisms of several typical SSDRC materials are systematically introduced.
- Based on the latest research, this review highlights the innovative applications in personal thermal management, outdoor building cooling, and energy harvesting, while also discussing the challenges and prospects for the future development of daytime radiative cooling.

## Introduction

Human activities and technological advancements have culminated in persistent global warming, a challenge that is anticipated to intensify in the coming decade [1]. The quest for effective cooling solutions has emerged as a critical global priority among sustainability experts. Traditional cooling technologies, predominantly air conditioning systems, not only consume substantial electricity, thereby contributing to heightened greenhouse gas emissions, but also exacerbate global warming due to the use of refrigerants. Consequently, there is an urgent need to identify innovative green cooling technologies. Daytime radiative cooling (DRC) is emerging as a passive cooling technique without any energy consumption [2–4]. This approach facilitates the dissipation of thermal radiation from objects into the universe via the atmospheric transmission window (ATW) while simultaneously rejecting solar irradiation to reduce photothermal load [5–7]. Heat escapes from surfaces as thermal radiation into the cold vacuum of space, resulting in a spontaneous temperature drop.

A diverse array of materials, including polymers [8–10], ceramic particles [11–13], photonic crystals [14–17], metamaterials [18–20], and fibrous materials [10, 21–23], have been reported for DRC applications. These materials usually present broadband-emissive characteristics due to their non-selective emissivity within the mid-infrared (MIR) wavelength range. Hence, the high non-ATW emissivity of MIR broadband-emissive materials results in the absorption of excessive thermal radiation from the surrounding environment, which affects their daytime radiative cooling. Spectrally selective daytime radiative cooling (SSDRC) materials are characterized by their dominant emission in the ATW wavelength bands while exhibiting low emission in the non-ATW ranges [24, 25]. Notably, SSDRC materials have three typical spectrum properties: (1) high reflectivity in the solar wavelength range to minimize sunlight absorption and mitigate photothermal effect; (2) high emissivity in the ATW range to facilitate thermal radiation dissipation into outer space; and (3) low emissivity in the non-ATW wavelength ranges to reduce thermal radiation absorption from the surrounding environment. In recent years, numerous reviews have summarized the principles [5, 26], materials [27–29], fabrication methods [2, 30, 31], and practical applications [28, 30] of DRC materials, offering valuable guidance for the development of passive cooling technology. Zhou et al. presented a comprehensive and systematic review of the fundamental design principles and fabrication methods for flexible photonic radiative cooling films, highlighting their potential in diverse applications such as building facades, photovoltaic devices, water harvesting, energy generation, environmental protection, and personal thermal management [32]. Nevertheless, the review did not address the critical aspects of spectrum-selective emission and corresponding design strategies. Recently, Hsu et al. reported a laminated SSDRC fabric [25]. This SSDRC fabric consisted of a polymethylpentene nanofibrous film, silver nanowires, and wool fabric. polymethylpentene nanofibrous film exhibited high solar reflectivity and selective emission properties with high emissivity limited to the ATW region. Silver nanowires provide high reflectivity across the entire MIR region to prevent infrared radiation from the surroundings to the human body. The wool fabric served as a broadband emitter, which absorbed thermal radiation from human skin through the textile-skin air gap and further conducted heat to the top PMP fabric via the silver nanowire layer. The nano–micro-hybrid laminated structure design and spectrum-selective properties of this SSDRC fabric offered superior body cooling performance compared to conventional non-selective DRC materials in hot outdoor scenarios [25, 33].

**Fig. 1:**
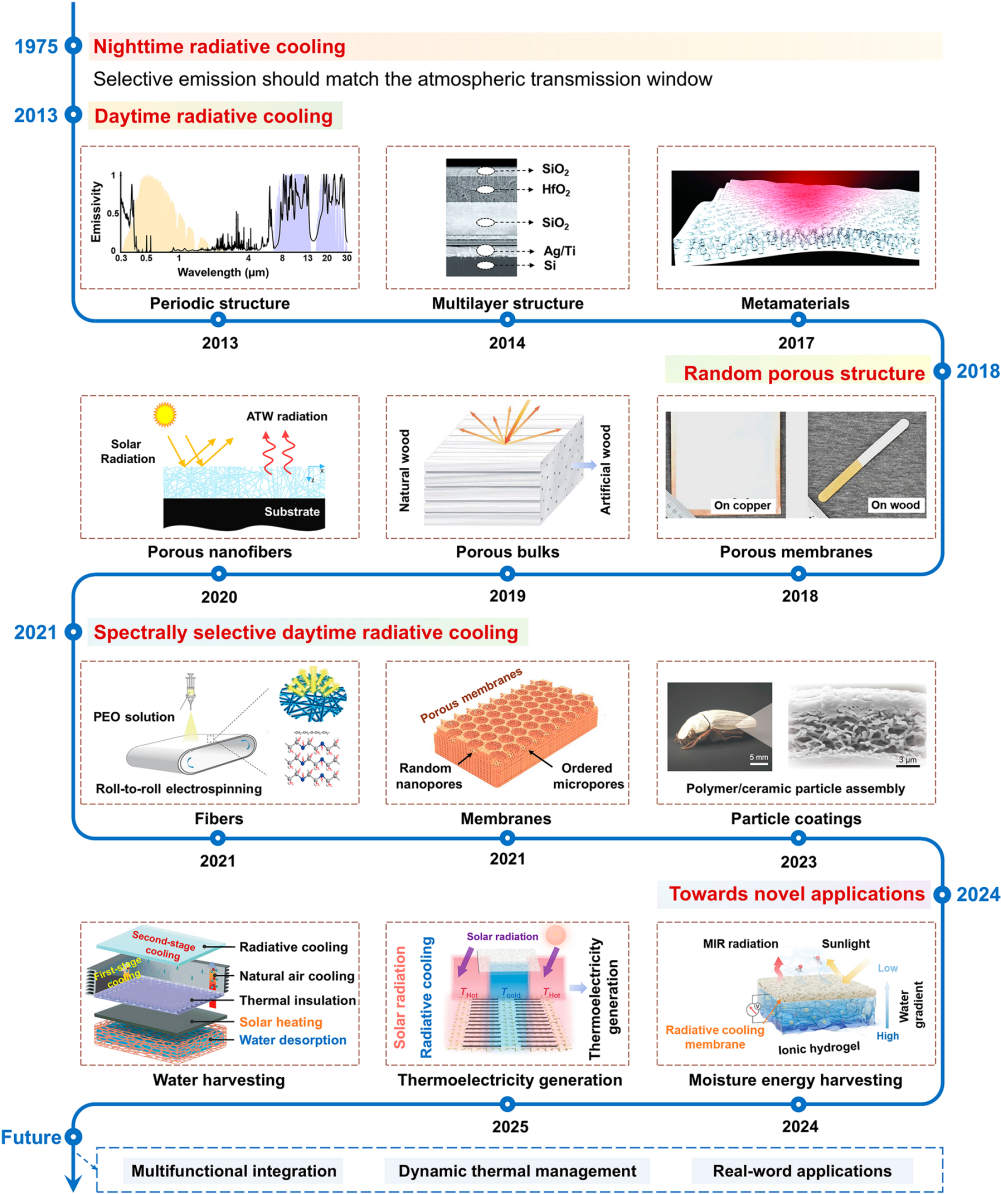
Materials and structure development of radiative cooling technology. Reproduced with permission from [35]. Copyright 2013, American Chemical Society. Reproduced with permission from [7]. Copyright 2014, Springer Nature. Reproduced with permission from [18]. Copyright 2017, AAAS. Reproduced with permission from [36]. Copyright 2018, AAAS. Reproduced with permission from [39]. Copyright 2019, AAAS. Reproduced with permission from [40]. Copyright 2020, Elsevier. Reproduced with permission from [10]. Copyright 2021, Springer Nature. Reproduced with permission from [132]. Copyright 2021, Springer Nature. Reproduced with permission from [44]. Copyright 2023, AAAS. Reproduced with permission from [52]. Copyright 2025, AAAS. Reproduced with permission from [191]. Copyright 2024, Springer Nature. Reproduced with permission from [51]. Copyright 2024, Royal society of chemistry

The general roadmap for the advancement of radiative cooling technology is illustrated in Fig. 1. The concept of radiative cooling was proposed over a century ago by Catalanotti et al. achieving nighttime radiative cooling by designing high-emissivity materials, particularly in the wavelength range of ATW [34]. Achieving daytime radiative cooling below ambient temperature remains challenging due to the photothermal effects of solar radiation, which often outweighs the outgoing thermal radiation to space. The first theoretical design for daytime radiative cooling was proposed by Raman et al. in 2013 [35]. Subsequently, they experimentally developed a DRC cooler using an integrated photonic solar reflector and thermal emitter in 2014 [7]. Since then, DRC has expanded greatly. Yin et al. reported a metamaterial membrane composed of a polymer layer embedded with silicon dioxide microspheres, backed by nanothickness silver [18]. This metamaterial membrane exhibited high daytime radiative cooling, leveraging the high emissivity of silicon dioxide microspheres within the ATW region and the high reflectivity of silver. Early DRC materials necessitate meticulous fabrication processes, often involving nanoscale and periodic-structure processing. These fabrication methods are intricate, relying on techniques such as photolithography and electron beam evaporation. Subsequently, with the discovery of many MIR selective-emissive materials [10, 18, 36–38], the development of DRC materials has seen significant progress, with a particular focus on the optimization of their solar reflectivity through the design of randomly arranged structures. Various random porous materials, including porous membranes [36], bulk materials [39], and nanofibrous materials [40], have been extensively studied. The random porous architecture of these materials enhances broadband sunlight scattering through Mie scattering, significantly improving their solar reflectivity. The materials and structure design of SSDRC materials were first explored by Zhu et al. [10]. They theoretically calculated that an ideal selective emitter could achieve a larger temperature drop than a non-selective emitter. They fabricated a polyethylene oxide nanofiber film with selective emission in the MIR range and experimentally verified its cooling advantages. Subsequently, a range of SSDRC materials, including fibers [10, 41], membranes [42], and ceramic particles [43, 44], were developed in succession. Advances in materials and photonic structure design have enabled SSDRC materials to achieve nearly perfect solar reflectivity. For instance, Tso et al. prepared a cellular ceramic composed of anisotropic porous networks [44]. This ceramic features a biomimetic porous structure, which efficiently scatters solar irradiation to achieve a sunlight reflectivity of 99.6%. With the rapid development of SSDRC technology, recent efforts have focused on novel applications such as electricity generation [45–47], electronic skin technology [48–50], and water harvesting [51]. Notably, our group presented large-area thermoelectric fabrics based on carbon nanotubes thermoelectric arrays and poly(vinylidene fluoride-co-hexafluoropropylene) (PVDF-HFP) SSDRC membranes, addressing thermal management and electricity generation in self-powered wearable applications [52]. Moving forward, the prospects of SSDRC emphasize multifunctional integration, dynamic thermal management, and practical real-world applications. So far, a comprehensive overview of the materials, structures, and design principles used for making high-performance SSDRC materials, and their adaptive applications for various energy management applications remains absent in the field.

**Fig. 2:**
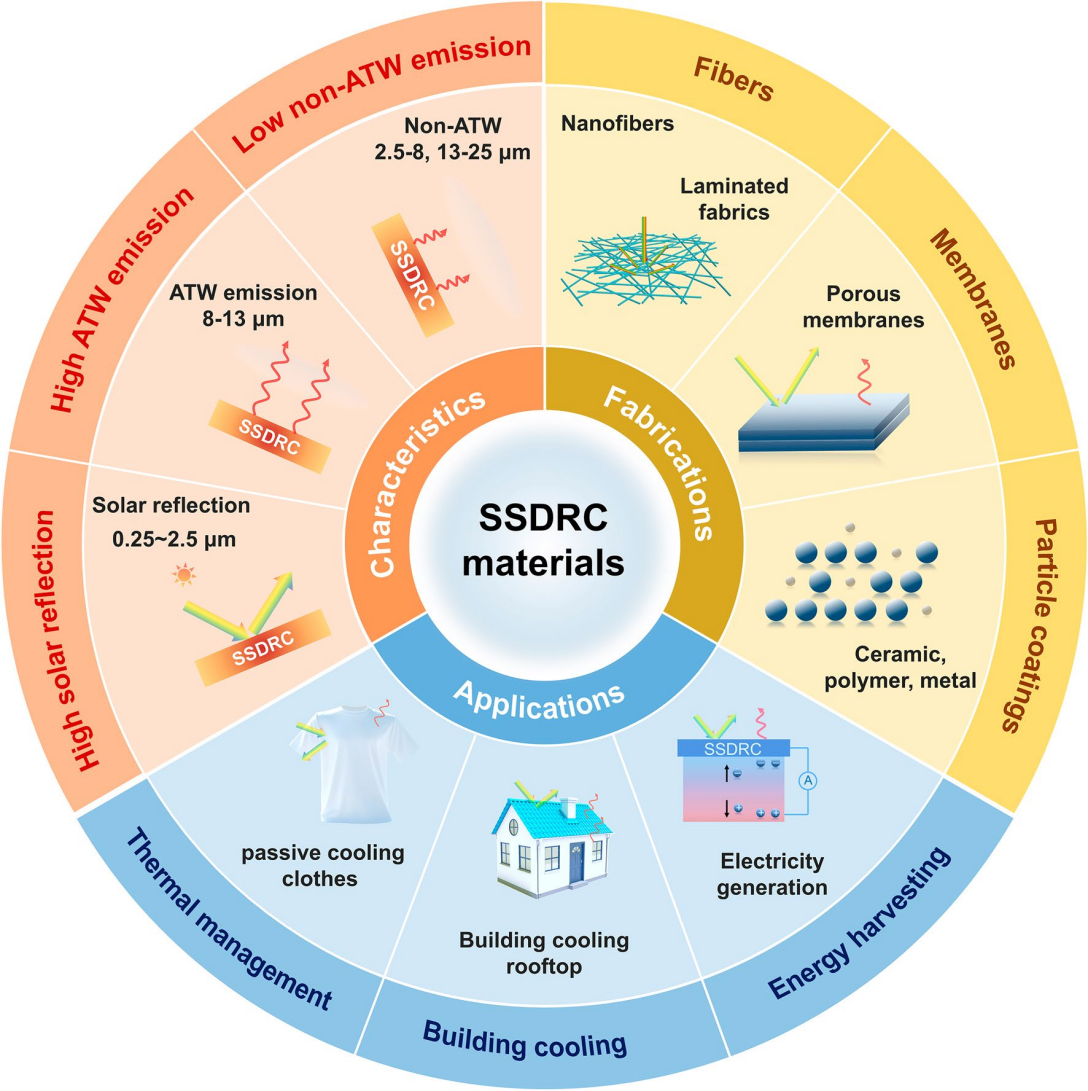
Scope of this review on the recent research progress in SSDRC materials

In this review, the design and fabrication of SSDRC materials and structures for highly efficient cooling and applications are discussed. As shown in Fig. 2, we first analyze the fundamental characteristics of SSDRC materials, including their molecular structures, micro- and nanostructures, optical properties, and thermodynamic principles. Subsequently, we comprehensively discuss the design and fabrication of three typical SSDRC materials, including fibers, membranes, and particle coatings, and address their optical properties and corresponding cooling mechanisms. Additionally, we review the practical applications of SSDRC materials in personal thermal management, outdoor building cooling, and energy harvesting. Finally, the remaining challenges and prospects associated with SSDRC materials are considered. By presenting the advancements in SSDRC materials and structures, the review aims to provide a comprehensive and updated reference, inspire innovative ideas, and advance the applications of radiative cooling technologies.

## Fundamental Characteristics of SSDRC Materials

The distinctive characteristic of SSDRC materials is their selective emission in the MIR wavelength range, compared with broadband emissive DRC materials. The spectrum-selective property in the MIR range is primarily attributed to the material’s optical control over light across various wavelengths, which is predominantly determined by the molecular and micro-/nanostructures of SSDRC materials.

### Molecular Structures of SSDRC Materials

**Fig. 3:**
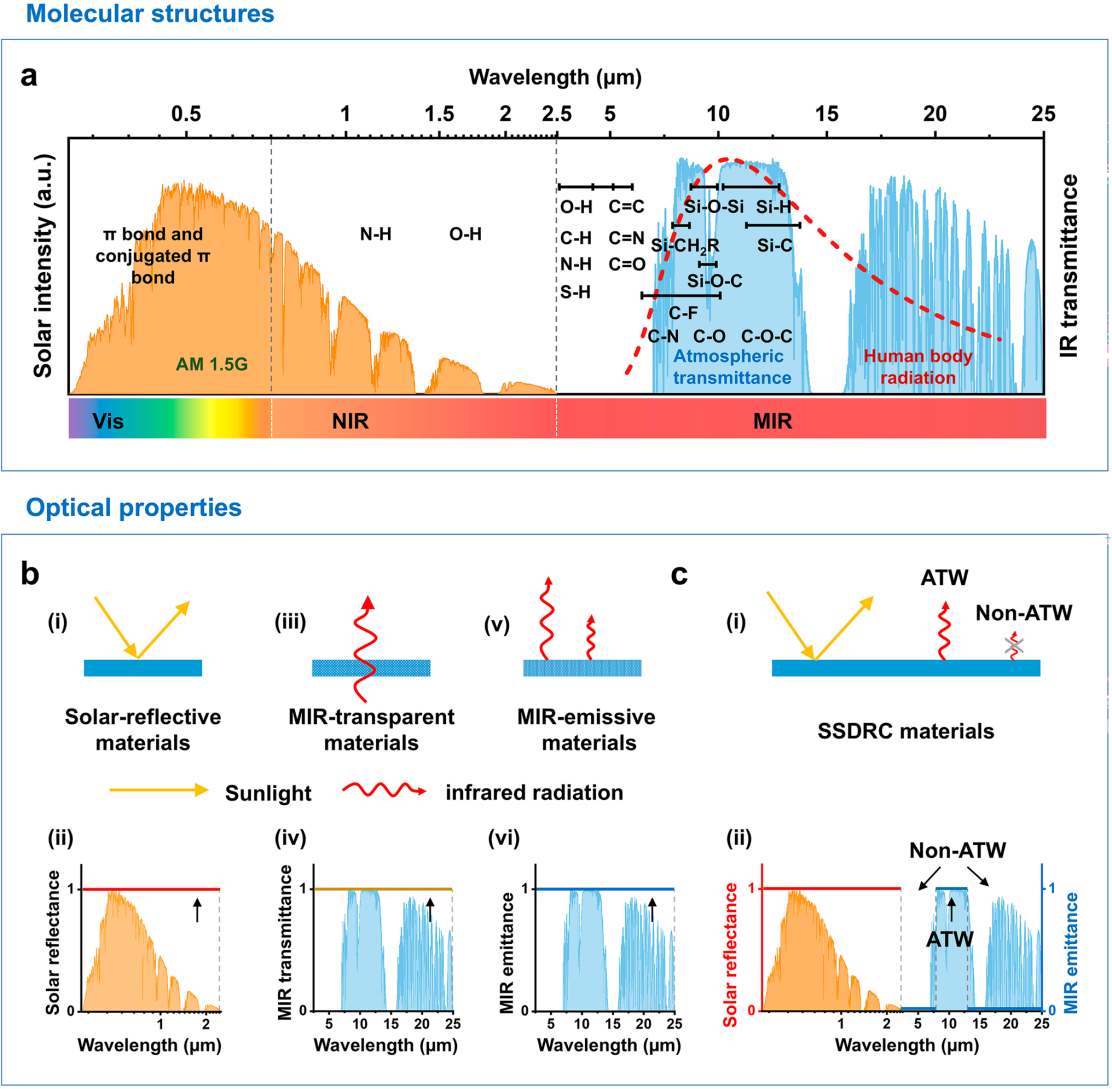
Molecular structures and optical properties of SSDRC materials. **a** Spectra of the partial chemical bonds and functional groups in the wavelength region from 0.25 to 25 μm. The normalized solar spectrum (AM1.5 G, orange shaded area), the atmospheric transmission spectrum (blue shaded area), and the human body radiation (red dashed line) are indicated as references. **b** Optical properties (i, iii, v) and the corresponding spectra (ii, iv, vi) of ideal solar-reflective materials, MIR-transparent materials, and MIR-emissive materials, respectively. **c** Optical properties (i) and the radiative spectrum (ii) of ideal SSDRC materials

The position of the infrared absorption peak is intrinsically related to the vibrational and rotational transitions of chemical bonds or functional groups within materials. Figure 3a illustrates the wavelength distribution of absorption peaks corresponding to the vibrational modes of various chemical bonds. Bending and stretching vibrations of chemical bonds generally occur within 400–4000 cm^−1^ (2.5–25 μm). Strong molecular vibrations in the ATW range are observed for Si–O–Si (8.3–10 μm), C–F (7.4–10 μm), S=O (9.4–9.8 μm), C–N (8.2–9.8 μm), C–H (11.1–14.3 μm), and C–O (7.6–9.5 μm), among others. These vibrations resonate with infrared waves, resulting in significant absorption/emission. Molecular vibrations associated with other chemical bonds or functional groups that fall outside the ATW region, such as N–H, O–H, C=C, C=N, amide, carboxyl, and benzene ring, have infrared emission in the non-ATW wavelength range. These characteristic molecular structures are unsuitable for SSDRC. Moreover, π bonds and conjugated π bonds exhibit strong absorbance of sunlight, which can lead to solar heating and adversely affect the radiative cooling effect.

### Micro- and Nanostructures of SSDRC Materials

To achieve deep sub-ambient cooling under direct sunlight, SSDRC materials must effectively reflect solar radiation and emit within the atmospheric transmission window. Consequently, the structures of SSRC materials must be designed to precisely control electromagnetic properties. Recent research reported that the electromagnetic properties of SSDRC materials can be manipulated through tailored micro-/nanostructures [2]. SSDRC materials reported to date can be categorized into three structures: multilayer structures [7, 53], metamaterials [54, 55], and random porous structures [56, 57]. Multilayer structures are composed of periodically stacked materials with different refractive indices (e.g., TiO_2_/SiO_2_), forming photonic bandgaps through Bragg scattering. This suppresses solar spectrum absorption while enhancing ATW thermal emission by wavelength-correlated photonic bandgap design [7, 35]. Their electromagnetic regulation mechanism originates from destructive interference caused by multiple reflections at the interfaces. Additionally, a bottom metal layer of Ag/Al can enhance UV–Vis reflection due to its high conductivity [58]. Although the photonic bandgap of such structures can be precisely tuned, their fabrication relies on high-precision deposition techniques like magnetron sputtering, resulting in relatively high manufacturing costs [59]. These structures are suitable for applications with stringent spectral selectivity requirements, such as thermal control systems in space [60]. The metamaterial is derived from the Greek language whose meaning is superior/beyond. Metamaterials are defined as artificially engineered composite structures or materials that exhibit extraordinary physical properties absent in naturally occurring substances [61–63]. Their fundamental characteristic lies in their ability to exhibit extraordinary physical properties unattainable in natural materials, including negative refractive index, negative permeability, negative permittivity and so on. Based on their target physical fields and application domains, metamaterials are systematically classified into electromagnetic metamaterials, acoustic metamaterials, mechanical metamaterials, thermal metamaterials and optical metamaterials [64]. Random porous structures enhance solar reflection through light scattering induced by nano-/microscale particles or pores [65]. Their electromagnetic mechanism relies on impedance mismatch-induced Rayleigh scattering and Mie scattering, while the molecular vibrational modes of porous polymers can simultaneously enhance atmospheric window emission. These structures are compatible with low-cost fabrication processes (e.g., spraying, phase separation, electrospinning), but their scattering efficiency is limited by particle size distribution. They are widely used in applications such as architectural coatings and smart textiles [10, 36, 43, 44].

### Optical Properties of SSDRC Materials

We discuss several cooling materials, including solar-reflective materials [66], MIR-transparent materials [67–69], MIR-emissive materials, and SSDRC materials, and compare their optical properties. As illustrated in Fig. 3b, solar-reflective materials exhibit high reflectance in the solar spectrum, effectively preventing the photothermal effect. Various solar-reflective materials have been developed for daytime cooling, including metals such as silver and aluminum, inorganic materials like silicon dioxide (SiO_2_), alumina (Al_2_O_3_), titanium dioxide (TiO_2_), zinc oxide (ZnO), and special photonic materials such as photonic crystal and optical metamaterial [70]. Szeto et al. demonstrated a cotton fabric with irregularly shaped TiO_2_ particle coatings as a solar reflector, which led to a temperature reduction of 3.91 °C [71]. Solar-reflective materials are primarily engineered to reflect solar radiation under direct sunlight, suggesting their effectiveness diminishes in other hot conditions. Solar-reflective materials are effective in reflecting solar radiation but cannot emit thermal radiation efficiently, limiting their cooling performance in non-sunlit conditions. In contrast, MIR-transparent materials exhibit high transmittance within the MIR wavelength range. Under typical indoor conditions, particularly at temperatures below that of the human body, radiation accounts for over 50% of the total heat loss of the human body [72]. Consequently, thermal radiation from the human body can be effectively emitted outside through MIR-transparent materials. For instance, Cui et al. reported a MIR-transparent nanoporous polyethylene (nano-PE) film [69]. This film featured interconnected pores that are 50 to 1000 nm in diameter, comparable in size to the wavelength of visible light, which strongly scattered visible light and made nano-PE opaque to human eyes. Moreover, the pore sizes were much smaller than the IR wavelength, so the nano-PE film remained highly transparent to mid-infrared light. The average transmittance of nano-PE was 96%, and it exhibited a superior cooling effect under indoor conditions. Compared to nano-PE films, PE yarns demonstrate superior advantages in personal thermal management applications due to their superior breathability and flexibility. Boriskina et al. fabricated PE fibers, yarns, and fabrics through melt spinning and weaving [73]. The resulting PE fabrics exhibited radiative cooling properties and excellent stain resistance, offering promise in reducing energy and water consumption during their operational phase. Currently, MIR-transparent materials are not engineered to reflect sunlight. The selection of materials for MIR-transparent radiative cooling requires a reduction in the content of functional groups that absorb sunlight.

MIR-transparent materials are effective for indoor human body cooling; however, implementing radiative cooling in outdoor applications, particularly for cooling objects, poses significant challenges. Currently, substantial research efforts are directed toward MIR-emissive cooling materials for outdoor passive radiative cooling. In general, high MIR-emissive materials are combined with high solar emissivity to maximize daytime radiative cooling efficiency. Hu et al. reported an artificial cooling wood [38]. This innovative material was produced through complete delignification and compression processes, resulting in a nanocellulose structure capable of effectively backscattering sunlight and strongly emitting in the MIR region, thereby achieving a DRC effect. Calculations indicated that the use of this cooling wood in construction could lead to savings of 20–60% in energy consumption.

MIR-emissive materials, while effective in broad-spectrum thermal emission, often lack the spectral selectivity required to maximize radiative cooling efficiency during daytime. In contrast, SSDRC materials combine high solar reflectivity with selective MIR emissivity within the atmospheric transmission window, enabling them to achieve efficient daytime radiative cooling. This dual functionality makes SSDRC materials uniquely suited for both solar reflection and thermal emission, addressing the limitations of other cooling materials. In detail, the spectrum is divided into three segments: the solar wavelength range (0.25–2.5 µm), the ATW wavelength range (8–13 µm), and the non-ATW wavelength range (2.5–8 µm and 13–25 µm). As illustrated in Fig. 3c, SSDRC materials possess high solar reflectivity, high ATW emissivity, and low non-ATW emissivity. SSDRC materials are engineered to reflect solar radiation while selectively emitting thermal radiation within the ATW range, where the atmosphere is most transparent. This minimizes heat absorption and maximizes heat dissipation. The low emissivity outside the ATW range further reduces unwanted heat exchange with the environment, enhancing cooling efficiency. These properties make SSDRC materials ideal for outdoor applications, where both solar reflection and thermal emission are critical for effective cooling. Table 1 provides a comprehensive comparison of the optical properties and cooling mechanisms of different cooling materials. Solar-reflective materials demonstrate high solar reflectivity, rendering them highly efficient for cooling in outdoor environments under intense sunlight. However, they exhibit limited cooling capabilities in indoor or non-sunlight conditions. Conversely, MIR-transparent materials enable the radiation of human body heat into the surrounding environment, making them particularly suitable for indoor or low-light scenarios. Broadband MIR-emissive materials, which dissipate human body heat through the atmospheric transmission window, are frequently integrated with solar-reflective materials to substantially improve outdoor cooling performance. In contrast, SSDRC materials synergize high solar reflectivity with selective MIR emissivity within the ATW. This combination not only enhances solar reflectivity but also reduces the heat load in the non-ATW range compared to broadband MIR-emissive materials, thereby achieving superior cooling performance.

**Table 1:** Optical properties and cooling mechanisms of different cooling materials

Material types	Optical properties	Cooling mechanism	Application scenario	Typical materials
Solar-reflective materials	High solar reflectivity	Reflect solar irradiation	Outdoor sunny condition	Metals (Ag, Ti, Al, etc.) [217], natural compounds (e.g., chlorophyll), photonic crystals [18, 70]
MIR-transparent materials	High mid-infrared transmittance	Thermal radiation emitted by the human body directly transmits to the environment	Indoor scene and cloudy day	MIR-transparent nano-PE microfibers and nano-PE membranes [69]
Broadband MIR-emissive materials	High mid-infrared emissivity	Emit human body thermal radiation to outer space	Outdoor condition	Natural polymers like silk [111], wool [218], cellulose [219]
SSDRC materials	High solar reflectivity, high ATW emissivity, low non-ATW emissivity	Reflect solar irradiation and selectively emit thermal radiation	Outdoor and sunny condition	As presented in Table 3

An ideal SSDRC material would exhibit 100% ATW emissivity and zero non-ATW emissivity. To quantitatively evaluate the spectrum selective of different SSDRC materials, the selective ratio, *γ*, was chosen as a quality factor to evaluate the MIR-emissive selection of SSDRC materials. *γ* is defined as the ratio of the average emissivity within the ATW to the emissivity within the non-ATW [25]. For typical radiative cooling applications, *γ* is expressed as Eq. 1:

1 $$\gamma = \frac{{\left( {\mathop \smallint \nolimits_{{8 \mu {\text{m}}}}^{{13 \mu {\text{m}}}} \varepsilon \left( \lambda \right){\text{d}}\lambda } \right)/\left( {\mathop \smallint \nolimits_{{8 \mu {\text{m}}}}^{{13 \mu {\text{m}}}} {\text{d}}\lambda } \right)}}{{\left( {\mathop \smallint \nolimits_{{2.5 \mu {\text{m}}}}^{{8 \mu {\text{m}}}} \varepsilon \left( \lambda \right){\text{d}}\lambda + \mathop \smallint \nolimits_{{13 \mu {\text{m}}}}^{{25 \mu {\text{m}}}} \varepsilon \left( \lambda \right){\text{d}}\lambda } \right)/\left( {\mathop \smallint \nolimits_{{2.5 \mu {\text{m}}}}^{{8 \mu {\text{m}}}} {\text{d}}\lambda + \mathop \smallint \nolimits_{{13 \mu {\text{m}}}}^{{25 \mu {\text{m}}}} {\text{d}}\lambda } \right)}}$$

where *ε(λ)* represents the emissivity at wavelength *λ*.

However, for ultra-high-temperature objects, the definition of selective ratio *γ* is different. Wien’s displacement law describes the spectral characteristics of thermal radiation, revealing an inverse relationship between blackbody temperature and peak emission wavelength. The law exhibits the product of the blackbody’s absolute temperature (*T*) and its spectral radiation peak wavelength (*λ*_max_) is a constant, expressed mathematically as Eq. 2 [74]:

2 $$\lambda_{\max } \times T = b$$

where *b* is the Wien’s displacement constant (0.897 µm K). This law indicates that the peak wavelength of thermal radiation shifts toward shorter wavelengths as the blackbody temperature increases. This shift highlights the importance of the 3–5 μm wavelength range for radiative cooling in extreme conditions. Specifically, for blackbodies with temperatures exceeding 331 °C, the proportion of thermal radiation within the 3–5 μm range surpasses that within the 8–13 μm range, despite the narrower bandwidth of the former [75]. To account for this phenomenon in ultrahigh-temperature environments, such as those encountered in arid regions, hot regions, and aerospace applications, the definition of spectral selectivity is modified as Eq. 3:

3 $$\gamma = \frac{{\left( {\mathop \smallint \nolimits_{{3 \mu {\text{m}}}}^{{5 \mu {\text{m}}}} \varepsilon \left( \lambda \right){\text{d}}\lambda + \mathop \smallint \nolimits_{{8 \mu {\text{m}}}}^{{13 \mu {\text{m}}}} \varepsilon \left( \lambda \right)d\lambda } \right)/\left( {\mathop \smallint \nolimits_{{3 \mu {\text{m}}}}^{{5 \mu {\text{m}}}} {\text{d}}\lambda + \mathop \smallint \nolimits_{{8 \mu {\text{m}}}}^{{13 \mu {\text{m}}}} {\text{d}}\lambda } \right)}}{{\left( {\mathop \smallint \nolimits_{{2.5 \mu {\text{m}}}}^{{3 \mu {\text{m}}}} \varepsilon \left( \lambda \right){\text{d}}\lambda + \mathop \smallint \nolimits_{{5 \mu {\text{m}}}}^{{8 \mu {\text{m}}}} \varepsilon \left( \lambda \right){\text{d}}\lambda + \mathop \smallint \nolimits_{{13 \mu {\text{m}}}}^{{25 \mu {\text{m}}}} \varepsilon \left( \lambda \right){\text{d}}\lambda } \right)/\left( {\mathop \smallint \nolimits_{{2.5 \mu {\text{m}}}}^{{3 \mu {\text{m}}}} {\text{d}}\lambda + \mathop \smallint \nolimits_{{5 \mu {\text{m}}}}^{{8 \mu {\text{m}}}} d\lambda + \mathop \smallint \nolimits_{{13 \mu {\text{m}}}}^{{25 \mu {\text{m}}}} {\text{d}}\lambda } \right)}}$$

The selective ratio γ and optical properties of some polymers and fabrics are listed in Table 2. PMP contains only C–C (954 to 1004 cm^−1^), -CH_2_ (1176 to 1241 cm^−1^), –CH (862 to 881 cm^−1^), and –CH_3_ (931 cm^−1^) bonds, leading to MIR absorption exclusively within the atmospheric transmission window range. Consequently, PMP displays a high selectivity ratio of 2.34, followed by PEO, PVDF-HFP, POM, and PP. Moreover, the molecular structures of materials like PET, cotton, silk, and wool, are predominantly composed of benzene rings and amino acids, whose infrared absorption peaks fall outside the atmospheric transmission window. As a result, they exhibit a selectivity ratio close to 1, highlighting their broadband-emissive properties.

**Table 2:** Emissive properties and selective ratios of typical radiative cooling materials [25]

Materials ^(a)^	Molecular structures	Attributes	Average ATW emissivity (%)	Average non-ATW emissivity (%)	Selective ratio
PMP	C–C, –CH, –CH_2_, –CH_3_	SP ^(b)^	85	15	2.34
PEO	C–C, –CH_2_, –CO	SP	90	30	1.9
PVDF-HFP [36]	C–C, –CF, –CF_2_	SP	97	50	/
POM [41]	–CH_2_, –CO	SP	75.9	~ 27	/
PP	C–C, –CH, –CH_2_, –CH_3_	SP	92	40	1.65
PI	–CONH–, –COOH, –NH_2_	SP	92	90	1.15
PA	–CONH–, –NH_2_	SP	95	94	1.05
PE	C–C, CH	SP	30	30	0.85
PET	C = O, C–O–C, –CH_2_, benzene ring	SP	85	80	1.21
Silk	amino acids, hydrogen bond, etc.	NP ^(c)^	95	95	1.01
Wool	amino acids, hydrogen bond, etc.	NP	95	95	1
Cotton	–OH, CH, –COO, benzene ring, hydrogen bond, etc.	NP	95	95	1.01

^a^*PMP* polymethylpentene, *PEO* polyethylene oxide, *PVDF-HFP* polyvinylidene fluoride-hexafluoropropene, *POM* polyoxymethylene, *PP* polypropylene, *PI* polyimide, *PA* polyamide, *PE* polythene, *PET* polyethylene terephthalate

^b^*SP* synthetic polymer

^c^*NP* natural polymer

### Thermodynamic Concepts of SSDRC Materials

**Fig. 4:**
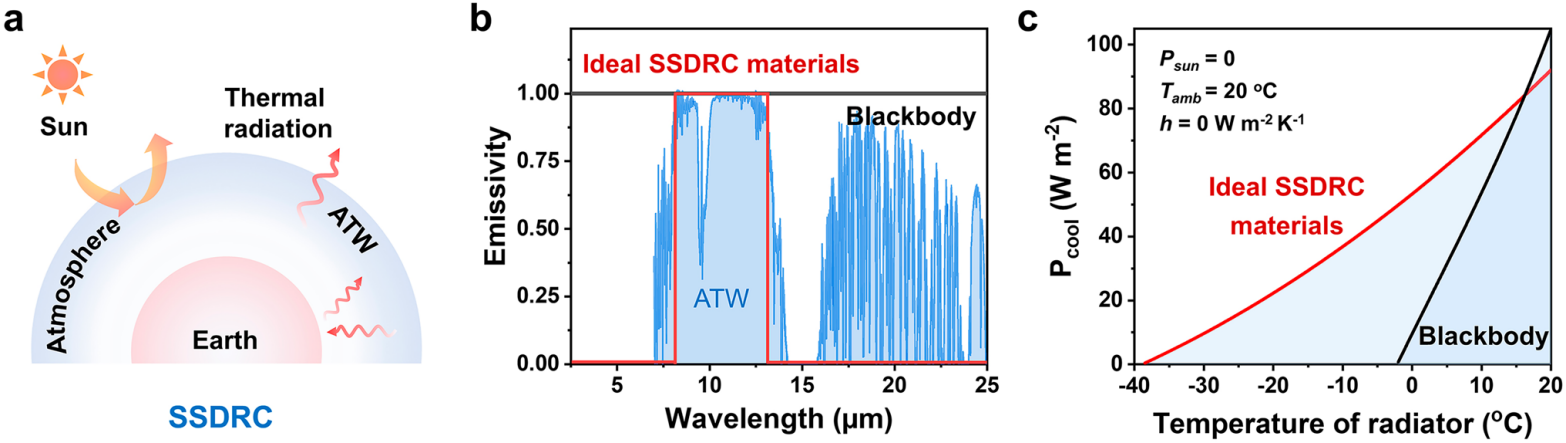
Cooling power calculation of ideal SSDRC materials. **a** Schematic of the radiative heat transfer process of SSDRC materials. **b** MIR-emissive spectra of ideal SSDRC materials and the blackbody. The background is the atmospheric transmission window. **c** Cooling power curves of the radiative cooler as a function of their temperatures, with the black curve representing the blackbody, and the red curve representing ideal SSDRC materials

As shown in Fig. 4a, SSDRC materials exhibit high solar reflectance and selective MIR emission in the ATW region. To highlight the cooling effect of ideal SSDRC materials, we calculate and compare their cooling power with that of non-selective materials. Cooling power is selected as the primary parameter for evaluating the performance of radiative coolers. The net cooling power is calculated by Eq. 4.

4 $$p_{{{\text{cool}}}} = P_{{{\text{emi}}}} - P_{{{\text{sun}}}} - P_{{{\text{atm}}}} - P_{c}$$

5 $$P_{{{\text{emi}}}} \left( T \right) = A\int {{\text{d}}\Omega \cos \theta } \mathop \smallint \limits_{0}^{\infty } {\text{d}}\lambda I_{{{\text{BB}}}} \left( {T,\lambda } \right)\varepsilon \left( {\lambda ,\theta } \right)$$

*P*_emi_ is the power emitted from the emitter. Here, *Ω* is a solid angle, *θ* denotes the angle between the direction of the solid angle and the normal direction of the surface, *ε*(*λ*,* θ*) is the emissivity of the object at a wavelength *λ* and angle *θ*, and *I*_BB_(*T*,* λ*) is the spectral irradiance of a blackbody.

6 $$P_{{{\text{atm}}}} \left( {T_{{{\text{atm}}}} } \right) = A\int {{\text{d}}\Omega \cos \theta } \int\limits_{0}^{\infty } {{\text{d}}\lambda I_{{{\text{BB}}}} \left( {T_{{{\text{atm}}}} ,\lambda } \right)} \varepsilon_{{{\text{atm}}}} \left( {\lambda ,\theta } \right)\varepsilon \left( {\lambda ,\theta } \right)$$

*P*_atm_ is the downward radiation from the atmosphere that is absorbed by the radiative cooler. *ε*_atm_(*λ*,* θ*) is the emissivity of the atmosphere.

7 $${P}_{c}=Ah\left({T}_{\text{atm}}-T\right)$$

*P*_*c*_ is the heat load from the conductive and convective heat exchange with the environment. *h* is a combined non-radiative heat transfer coefficient.

As illustrated in Fig. 4b, a blackbody exhibits a broadband emission within the wavelength range of 3–25 µm, with an emissivity of 100%. In contrast, an ideal SSDRC material displays emission only within the 8–13 µm range, with zero emissivity outside this interval. We compare the cooling power of the ideal SSDRC with that of the blackbody. Cooling powers *P*_cool_ as a function of the temperatures are calculated using Eq. 4. Here, we assume an ambient temperature *T*_atm_ of 20 °C, zero solar absorption (*P*_sun_ = 0), and thermal insulation with the environment (*h* = 0). As illustrated in Fig. 4c, when the temperature of an object substantially falls below the ambient temperature, the cooling power of ideal SSDRC material markedly surpasses that of the blackbody, indicating its superior passive cooling performance. The shaded area in Fig. 4c delineates the material’s radiative cooling range, demonstrating that SSDRC exhibits a more pronounced radiative cooling effect across a broader temperature range compared to the blackbody.

## Design and Fabrication of SSDRC Materials

The design and preparation of SSDRC materials involves utilizing MIR spectral selective materials as primary components, which are then designed to achieve high solar reflectivity and thus enable spectral selection over the entire spectral range. Recent reviews have reported many methods for regulating the reflectivity of solar light. The optical properties of SSDRC materials within the solar spectrum can be tailored through engineered micro-/nanostructures [2], which primarily include multilayer structures [76–78], metamaterial structures [19, 78, 79], and porous structures [42, 80–82]. The optical manipulation of these structural materials is based on principles of photonic bandgap [14, 83, 84], Fabry–Pérot resonance [85], and random scattering [78, 86–88]. When light encounters an object, it interacts with its atoms or molecules, leading to scattering in various directions. This scattering phenomenon is influenced by refractive index differences, surface irregularities, or particle sizes. Mandal et al. demonstrated through FDTD simulations that a broadly distributed range of pore sizes, from 50 nm to 5 μm, can effectively scatter the entire solar spectrum [36]. Through randomly distributed micro/nanoparticles or pores, a broad light-scattering effect is generated, resulting in high solar reflectivity. Fibrous materials, porous membranes, and particle coatings have high solar reflectivity due to their capacity for random sunlight scattering. In this section, we primarily discuss three typical types of SSDRC materials: fibers, membranes, and particle coatings. These materials represent classic and widely studied approaches in the field of radiative cooling. Beyond these three categories, other novel material types and designs, including fluorescent materials, hydrogels and bio-mass materials, are detailed in Table 3. This table comprehensively summarizes their unique properties, preparation methods, and technological innovations, providing a broader perspective on the diversity and advancements in SSDRC materials.

**Table 3:** Summary of various SSDRC materials, their preparation methods, optical properties, and cooling performances

Types/structures	Materials	Preparation methods	Optical properties		Cooling performances		Weather conditions	Innovations	References
			Solar reflectivity (%)	ATW emissivity (%)	Cooling power (W m^−2^)	Temperature drop (°C)			
Fibers	PVDF/PVA nanofibers	Electrospinning	94	94	–	9	Solar intensity: 900 W m^−2^	High thermal insulation	[107]
	PTFE/cellulose fibers	Air-spray	93	92	104	5	Solar intensity: 834 W m^−2^, ambient temperature: 30 °C	Self-cleaning performance	[220]
	Silk-based laminated fabrics	Hot pressing	96.5	97	–	5.1	Solar intensity: 892.4 W m^−2^	Moisture permeability, high mechanical strength	[111]
	PLA/silk fibers	Melt-blowing, electrospinning, and laminating	85	96	–	2.7	Solar intensity: 550 W m^−2^	Air filtration and radiative cooling	[221]
	PLA-TiO_2_/PTFE metamaterial fabrics	Melt spinning, weaving, and laminating	92.4	94.5	~ 50	4.8	Solar intensity: 600 W m^−2^, ambient temperature: 20 °C	Hierarchical morphology design	[98]
	Al_2_O_3_/silk fabrics	Reagent-assisted dip-coating	95	90	–	8	Solar intensity: 825 W m^−2^, ambient temperature: 35 °C	Molecular structure design	[108]
	PTFE/POM nanofibers	Electrospinning	95.4	83.2	151.8	9	Solar intensity: 820 W m^−2^, ambient temperature: 31 °C, relative humidity: 10%	Spectrally selective design	[24]
	PMP/wool/AgNW laminated fabrics	Electrospinning, lamination	97	85	46.3	6.2	Solar intensity: 1010 W m^−2^, ambient temperature: 34 °C	Molecular structure design	[25]
	PEO nanofibers	Electrospinning	96.3	78	80	5	Solar intensity: 800 W m^−2^, ambient temperature: 17 °C	All-day radiative cooling materials	[10]
Membranes	PDMS/Al membranes	Blade coating	–	94.6	120	9	/	Large-area manufacturing	[222]
	PDMS/Al membranes	Coating	92.1	94.5	52.4	2.4	Solar intensity: 750 W m^−2^, ambient temperature: 24 °C, wind speed: 1.5 m s^−1^, relative humidity: 40%	Sample and large-scale preparation methods	[223]
	Porous PVDF-HFP membranes	Phase inversion	96	97	96	6	Solar intensity: 880 W m^−2^, ambient temperature: 14.5 °C	Sample preparation	[36]
	Inverse opal PMMA membranes	Template method	95	98	85	5.5	Solar intensity: 930 W m^−2^, relative humidity: 64%	Low-cost, all-day cooling	[132]
	PDMS/PE membranes	Lamination method	96	80	70 ± 14	5–6	Solar intensity: ~ 990 W m^−2^, ambient temperature: 38 °C, relative humidity: 33%	Bioinspired design; switchable cooling and heating	[224]
	SiO_2_/TPX/Ag metamaterial membrane	Roll-to-roll, electron beam evaporation	96	93	93	–	–	High-throughput and economical preparation	[18]
	SiO_2_/TPX membranes	Solution processing method	< 10	85	–	5	Ambient temperature: 35 °C	Transparent radiative cooling and self-cleaning	[225]
	PS/PMMA membranes	Nanoimprinting, phase separation and blade coating	93.4	92.3	–	7.2	Solar intensity: ~ 800 W m^−2^, ambient temperature: 16 °C, wind speed: 2 m s^−1^, relative humidity: 35%	Brilliant colorful daytime radiative cooling	[17]
	PDMS/Ag transparent radiative cooler	Electron beam evaporation and photolithography	–	–	–	22.1	Solar intensity: ~ 600 W m^−2^, wind speed: 4.5 m s^−1^, relative humidity: 25%	Transparent radiative cooling	[226]
	Silica-coated aluminum oxide	Two-step anodization	86	96	–	6.1	Solar intensity: ~ 850 W m^−2^, ambient temperature: 33 °C, wind speed: 1.5 m s^−1^, relative humidity: 30%	Simple and low-cost preparation	[227]
Particle coatings	Colorant/PVDF-HFP bilayer coatings	Phase inversion and coating	89	96	–	15.6	Solar intensity: ~ 1025 W m^−2^	Colored radiative cooling materials	[200]
	Al_2_O_3_/SiO_2_	Blade coating and drop casting	94.1	93.5	100	7.9	Solar intensity: ~ 600 W m^−2^, ambient temperature: 30 °C, wind speed: 1 m s^−1^, relative humidity: 59%	Simple and low-cost preparation	[228]
	SiO_2_/ZnO/PMMA	Annealing, coating	96	94	–	4.1–5.3	Solar intensity: 800 W m^−2^, ambient temperature: 29 °C	Anti-fouling	[229]
	SiO_2_/BaSO_4_	Ball milling, spray	95	96	89.6	6.2–8.1	Solar intensity: ~ 600 W m^−2^, ambient temperature: 27 °C, relative humidity: 24%	Biomimetic skin structure	[230]
	SiO_2_/Al coatings	Spray coating	97	89	–	7.3–9.1	Solar intensity: ~ 600 W m^−2^, ambient temperature: 17 °C, wind speed: 2.5 m s^−1^	Sample preparation methods	[231]
	Al_2_O_3_/glass coatings	Coating	> 96	95	60	3.5–4	Solar intensity: ~ 750 W m^−2^, ambient temperature: 30 °C	Long-term environmental stability	[43]
	Al_2_O_3_/PES/NMP	Cast	99.6	96.5	130	4.3	Solar intensity: ~ 700 W m^−2^, wind speed: 4 m s^−1^, relative humidity: 30%	Near-perfect solar reflectivity, durability	[44]
Fluorescent materials	Silica-embedded perovskite nanocrystals	Solvothermal method	–	> 90	–	4.2	Ambient temperature: 12 °C, wind speed: 0.839 m s^−1^, relative humidity: 29%	Colored daytime radiative cooling	[232]
	Light-emitting perovskite nanocrystals	Solvothermal, and coating method	–	90	103.09	8.68	Solar intensity: 770 W m^−2^, ambient temperature: 35 °C	Colored daytime radiative cooling	[233]
	Cu-based quantum dots	Spray coating	–	~ 85	40.75	3.25	Solar intensity: ~ 850 W m^−2^, ambient temperature: ~ 30 °C	Colored daytime radiative cooling	[234]
	cellulose acetate/perovskite quantum dots	electrostatic-spinning/inkjet printing	–	–	25.6–51.7	2.2–5.4	Solar intensity: 740 W m^−2^, ambient temperature: ~ 38 °C, relative humidity: 40%	Colored daytime radiative cooling	[235]
Hydrogels	NPs/NADES@PAAm/PVA	Liquid casting	~ 95	~ 92	57	6.2	Solar intensity: 7220 W m^−2^, ambient temperature: 35 °C, humidity: 30%	Radiation and evaporation cooling	[236]
	poly(N-isopropylacrylamide-co-acrylamide) hydrogel	Phase separation	–	95	–	9.14	Solar intensity: ~ 2000 W m^−2^, ambient temperature: ~ 25 °C	Transparent cooling	[237]
	PNIPAm/PVDF-HFP	light-initiated free radical polymerization	70	96	47	1.8–3.7	Solar intensity: 500 W m^−2^, ambient temperature: 40 °C	Self-adaptive regulation	[238]
	bacterial cellulose aerogel/PVA hydrogel	solvent exchange, and dip-coating	98.8	86	–	~ 18	/	Facile fabrication	[239]
	BaSO_4_/PVA hydrogel	Mixing, crosslinking	88	–	350	4–8	Solar intensity: 1140 W m^−2^, relative humidity:60–80%	Suitable for high-humidity conditions	[240]
Bio-mass materials	Treated wood	Delignification, and mechanical pressing	–	–	53	9	Ambient temperature: 20 °C	All-weather cooling	[39]
	Protein/DNA aerogel	Bidirectional freeze-drying and water welding	> 90	> 90	–	16	Solar intensity: ~ 300 W m^−2^, ambient temperature: 29 °C	Have a visible-light reflectance exceeding 100%	[241]
	Sodium alginate aerogel	sol–gel, freeze-drying	90	98.7	–	11.5	Solar intensity: 1041 W m^−2^, ambient temperature: 31 °C, relative humidity: 65%	Multiscenario thermal management	[242]

### Fibers

Micro-/nanofibrous materials effectively scatter sunlight via their porous morphological structures and multiscale pore distributions [28]. Here, we discuss the fabrication, structures, and performance of SSDRC fibrous materials, focusing on nanofibrous materials, and laminated fabrics.

#### SSDRC Nanofibrous Materials

**Fig. 5:**
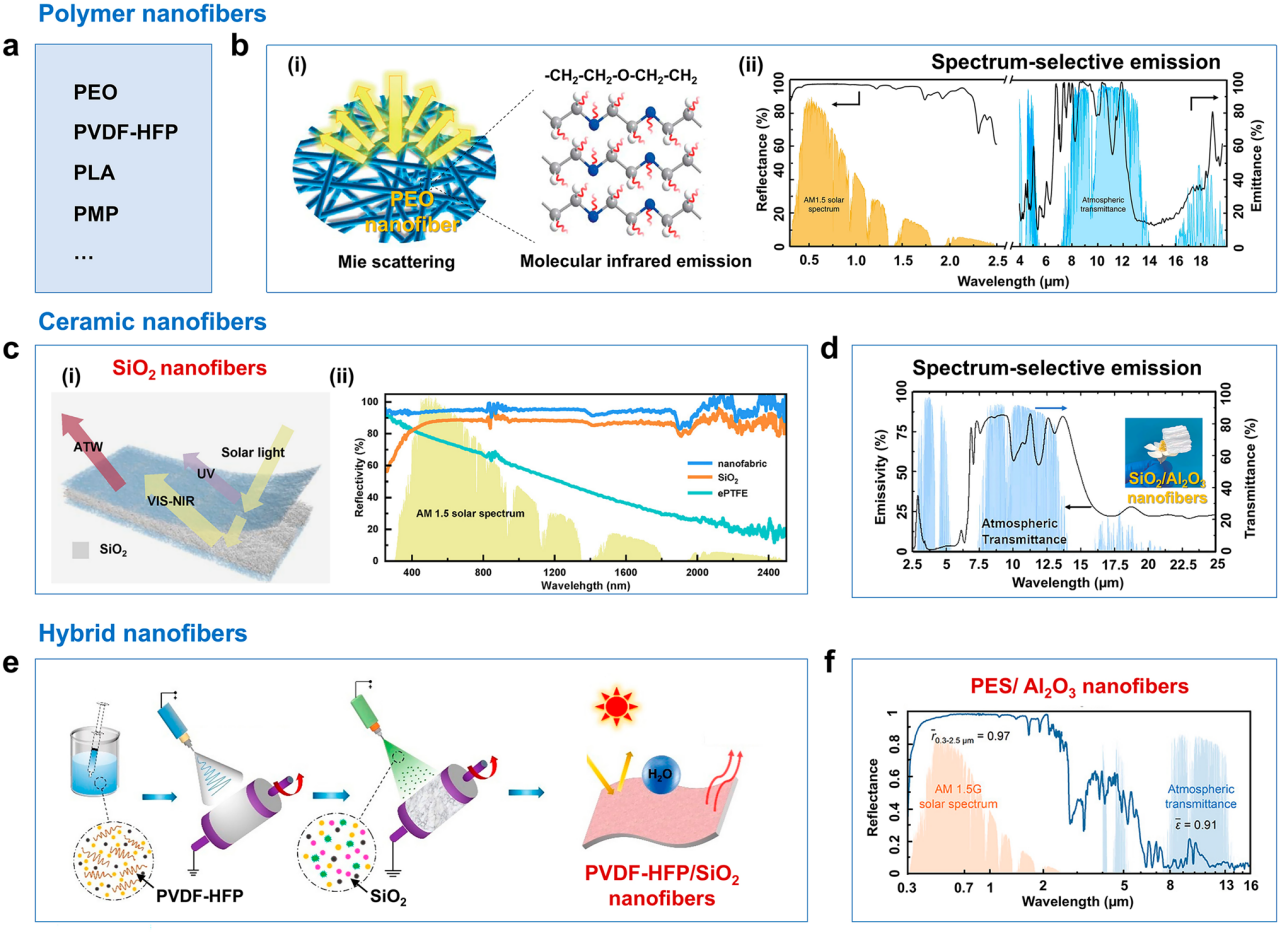
SSDRC nanofibrous materials. **a** Typical SSDRC polymer nanofibers. **b** PEO SSDRC nanofibers. (i) Schematic of infrared emission by C–O–C bond vibrations of PEO molecular chains and Mie scattering from micro-/nanostructures of PEO nanofiber film. (ii) Solar reflectance and MIR emission spectra of PEO nanofibers. Reproduced with permission from [10]. Copyright 2021, Springer Nature. **c** Schematic structure and solar reflectance spectra of SiO_2_ nanofibers. Reproduced with permission from [93]. Copyright 2024, Elsevier. **d** MIR emission spectrum (2.5–25 μm) of SiO_2_/Al_2_O_3_ nanofibers. Reproduced with permission from [101]. Copyright 2023, Elsevier. **e** Schematic fabrication of PVDF-HFP/SiO_2_ nanofibers. Reproduced with permission from [105]. Copyright 2022, American Chemical Society. **f** The reflectance spectrum (0.3–16 μm) of PES/Al_2_O_3_ nanofibers. Reproduced with permission from [106]. Copyright 2023, John Wiley and Sons

Electrospinning has emerged as a versatile technique for fabricating micro-/nanofibrous film, primarily due to its tunability in pore structure through adjustments of electric field parameters and solution properties (e.g., viscosity, conductivity) [89–91]. The electrospinning process, characterized by the interplay of electric fields, airflow, fiber entanglement, and solution attributes, produces fibers with random distributions. Such porous architecture is advantageous for sunlight reflection. Using Mie theory, the scattering efficiency of nanofibers as a function of diameter across the solar spectrum reveals that nanofibers with diameters ranging from 500 to 1200 nm can effectively scatter sunlight (especially in the 0.3–1.2 μm wavelength range, which covers the majority of the solar wavelength range) [10]. Currently, reported SSDRC nanofibrous materials include polymer nanofibers (PEO, PVDF-HFP, PLA, PMP, etc.) (Fig. 5a), ceramic nanofibers (SiO_2_, Al_2_O_3_, etc.), and hybrid nanofibers. A critical challenge lies in balancing the high light-scattering efficiency with the mechanical stability and environmental tolerance. For instance, while Zhu et al. demonstrated that PEO nanofibrous film (Fig. 5b) achieves 96.3% solar reflectivity through broad diameter distributions centered at 800 nm [10], this design relying on C–C/C–O bond-mediated selective absorption may face long-term chemical degradation under UV irradiation, a risk not explicitly addressed in the original study. In contrast, ceramic nanofibers (e.g., SiO_2_, Al_2_O_3_) exhibit superior photothermal stability but suffer from brittleness and high manufacturing costs, limiting their scalability. This dichotomy suggests that future research should prioritize organic–inorganic hybrid systems. This contrast indicates that future studies should focus on developing organic–inorganic hybrid systems. For example, combining PEO’s flexible structure with ceramic coatings could create materials that simultaneously enhance both optical properties and durability. Beyond electrospinning, a common method for nanofiber production, other techniques such as solution blow spinning [92–94], centrifugal spinning [95], wet spinning [96], microfluidic spinning [97], and melt spinning [98] are also used to create micro-/nanofibrous materials. These methods are particularly valuable for developing nanofiber films with radiative cooling capabilities. For example, Chen et al. fabricated a multilayered fabric through solution blow spinning, incorporating nylon 66 nanofibers, silver particles, and colloidal photonic crystal coatings [92]. This innovative design demonstrated significant passive radiative cooling performance, achieving a temperature reduction of 7.9 °C under direct solar irradiation. Melt spinning provides an effective method for uniformly incorporating radiative cooling micro/nanoparticles into polymer matrices through an integrated process of high-temperature melting, mechanical drawing, and rapid quenching to produce continuous filament fibers. Compared to non-woven fabrics produced by electrospinning or blow spinning, melt-spun filament fibers offer distinct advantages for textile applications, including superior mechanical properties for knitting and weaving processes and improved dye uptake and color fastness. A representative demonstration by Tao et al. [98] employed this technique to fabricate TiO_2_-PLA metafibers through a systematic process. First, TiO_2_-PLA composite materials were fabricated using a twin-screw extruder at a specific mass ratio and a high temperature of 205 °C. Then, post-cooling TiO_2_-PLA composite materials were spun to fabricate the metafibers using a melt spinning machine. Finally, the metafibers were stretched and collected on the draw winder machine. The TiO_2_-PLA metafibers exhibited superior tensile properties with an elongation of 29.5% and a breakage strength of 1.886 cN per decitex, which are flexible and strong enough to be stitched via a commercial sewing machine.

Ceramics offer superior resistance to thermal, ultraviolet radiation, and chemical corrosion compared to polymers, therefore, various SSDRC ceramic nanofibers are demonstrated [99–103]. However, their inherent brittleness and high fabrication costs remain significant barriers to widespread adoption. For instance, Zhang et al. achieved 94% solar reflectivity and 94% ATW emissivity in SiO_2_ nanofibrous films via blow spinning and annealing [93] (Fig. 5c). This method’s reliance on high-temperature calcination raises concerns about energy consumption and scalability. Similarly, Sun et al. addressed silica’s fragility by incorporating Al_2_O_3_ into electrospun nanofibers, forming a multilayer network that enhanced mechanical integrity and achieved 95% solar reflectivity with 5 °C sub-ambient cooling [101] (Fig. 5d). Yet, the trade-off between mechanical reinforcement and optical performance remains poorly understood. Increasing alumina content might accidentally lower porosity, which could decrease radiative cooling efficiency. Hybrid polymer/ceramic systems [104], such as PVDF-HFP/SiO_2_ [105] and polyethersulfone (PES)/Al_2_O_3_ [106] nanofibers (Fig. 5e and f), attempt to reconcile flexibility with durability. PVDF-HFP/SiO_2_ film leveraged molecular vibrations and phonon-polarization effects to achieve 95% MIR emissivity [105], yet its long-term stability under cyclic thermal stress remains unverified. Dual-mode textile exemplifies innovation in adaptive thermal management [106], combining a PES/Al_2_O_3_ cooling layer (97% solar reflectivity) with an MXene warming layer (85% solar absorptance). While this design demonstrates versatility, its complexity creates manufacturing challenges. The system requires the integration of different materials with opposing properties, specifically in terms of hydrophilicity and interfacial adhesion. Additionally, the MXene heater depends on electrical conductivity for Joule heating during low solar conditions. This energy requirement may restrict its use in off-grid applications.

#### SSDRC Laminated Fabrics

**Fig. 6:**
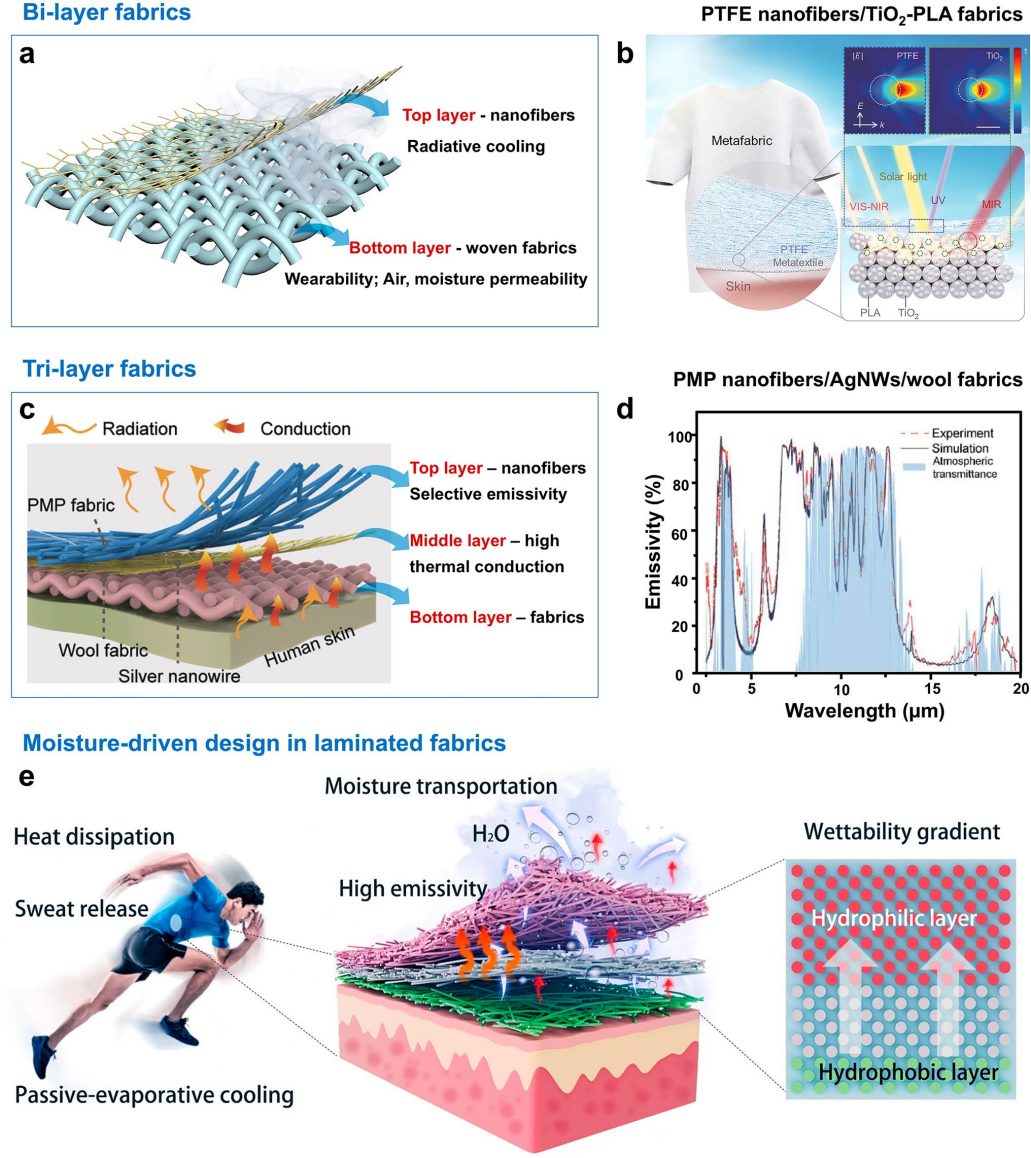
SSDRC laminated fabrics. **a** Schematic of the bilayer fabrics and functions of different layers. **b** Schematic of a PTFE/TiO_2_-PLA metamaterial fabric with a bilayer structure for daytime radiative cooling. Reproduced with permission from [98]. Copyright 2021, AAAS. **c** Schematic structures of the PMP/AgNW/wool tri-layer fabrics and the functions of different layers. **d** Experimental and theoretical results of the emissivity of top PMP nanofibers. Reproduced with permission from [25]. Copyright 2021, AAAS. **e** Schematic depicting the heat dissipation and sweat release process of WGID membrane. Reproduced with permission from [112]. Copyright 2024, Springer Nature

Laminated fabrics enhance the overall radiative cooling performance by combining different layers of fibers, utilizing the special optical and thermal properties of each layer to achieve spectral selection among the solar and MIR spectra [98, 107–110]. SSDRC laminated fabric includes bilayer fabrics and tri-layer fabrics. The double-layer fabrics are mainly composed of nanofibers and woven fabrics. As shown in Fig. 6a, nanofibers are typically used as spectrally selective materials and high solar reflectance materials, while the breathability and mechanical properties of the woven fabric make it suitable for application in the clothing industry. In addition, endowing the woven fabric with a metamaterial structure can further improve the radiative cooling performance of the bilayer fabric. For instance, Tao et al. designed a bilayer metamaterial fabric, knitted with composite microfibers that incorporated random metamaterial structures (Fig. 6b) [98]. The bottom layer consisted of a titanium oxide-polylactic acid (TiO_2_-PLA) woven fabric, which embodied nanobeads with diameters ranging from 200 to 1000 nm and nanofibers with lengths of several micrometers. The top layer was a 50-μm-thick polytetrafluoroethylene (PTFE) film, which effectively reflect ultraviolet light. Additionally, PLA microfibers, which have C=O, CH_3_, CH, C–O, and C–C chemical bonds, provided rich emittance in the MIR wavelength range. The hierarchical morphology design endowed the PTFE/TiO_2_-PLA metamaterial fabric with a solar reflectivity of 92.4% and an average ATW emissivity of 94.5% through hierarchical structures. While innovative, this design’s reliance on PTFE films raises concerns about breathability, as PTFE’s low moisture permeability may compromise wearer comfort in humid environments. The multistep fabrication processes of melt spinning, weaving, and lamination pose scalability challenges when compared to single-step electrospinning. This underscores the need to simplify manufacturing protocols to balance performance with industrial feasibility.

Tri-layer fabrics demonstrate superior potential for wearable radiative cooling compared to bilayer designs, primarily due to their capacity to integrate directional heat transfer mechanisms. As shown in Fig. 6c, the middle layer of PMP/AgNW/wool fabric is silver nanowires, which have high thermal conductivity and can quickly transfer the heat from the skin to the outer high-emissive PMP nanofibers. The PMP layer exhibited a high selective ratio of 2.23 and an average ATW emissivity of 0.85 [25]. PMP had a wide range of size distributions owing to the sequential volatilization of solvent during electrospinning, which enabled a wideband scattering efficiency that covers the entire solar spectrum. PMP only had C–C, –CH_2_, –CH, and –CH_3_ bonds, resulting in high absorption primarily in the ATW range (Fig. 6d). The bottom wool fabric absorbed thermal radiation emitted by the skin, which was then conducted through the AgNW to the surface PMP fabric for selective thermal emission, thereby enhancing the cooling capability. Zhang et al. developed a multilayer silk fabric consisting of three layers of fabric [111]. The multilayer silk fabric was composed of a PTFE film, a commercial silk fabric, and electrospun silk nanofibers. The electrospun silk nanofibers featured a hierarchical structure that enhanced sunlight scattering and offered remarkable thermomechanical stability. The PTFE film and commercial silk fabrics provided ultraviolet reflectance and mechanical strength. The excellent mechanical performance, surface hydrophobicity, and ultraviolet resistance endowed the multilayer silk fabric with outdoor durability, including high solar reflectance (96.5%) and MIR emittance (97.1%). SSDRC laminated fabrics allow precise control over the thickness, material composition, and optical properties of each layer, enabling highly tunable cooling performance. By selecting specific interlayer materials, SSDRC laminated fabrics can be endowed with multiple functionalities such as breathability, antifouling properties, and mechanical strength. The application of moisture-driven materials in laminated fabrics offers innovative and efficient solutions for personal thermal management [112–114]. Firstly, dynamic modulation of infrared emissivity can be achieved by regulating the moisture adsorption and release behavior of fabrics, thereby optimizing cooling performance. For instance, adaptive textiles based on Ti_3_C_2_T_x_ MXene enable infrared emissivity modulation from 12 to 68% through the intercalation and deintercalation of water molecules between layers [115]. This mechanism allows real-time adjustment of thermal radiation in response to environmental humidity or human sweat. Secondly, multilayer fabrics with diode-like unidirectional transportation properties, achieved through Janus wettability design, enhance sweat evaporation cooling and improve comfort. For example, Hu et al. developed a wettability-gradient-induced-diode (WGID) membrane using MXene-engineered electrospun technology (Fig. 6e) [112]. This membrane facilitates heat dissipation and moisture-wicking transportation, achieving a cooling temperature of 7.1 °C due to its high ATW emissivity of 96.4% and unidirectional moisture transportation properties.

### Membranes

Porous membranes can be categorized into different types, such as random porous membranes, ordered porous membranes, and gradient porous membranes. In this section, we discuss the preparation and impact of these porous structures on the performance of SSDRC materials.

**Fig. 7:**
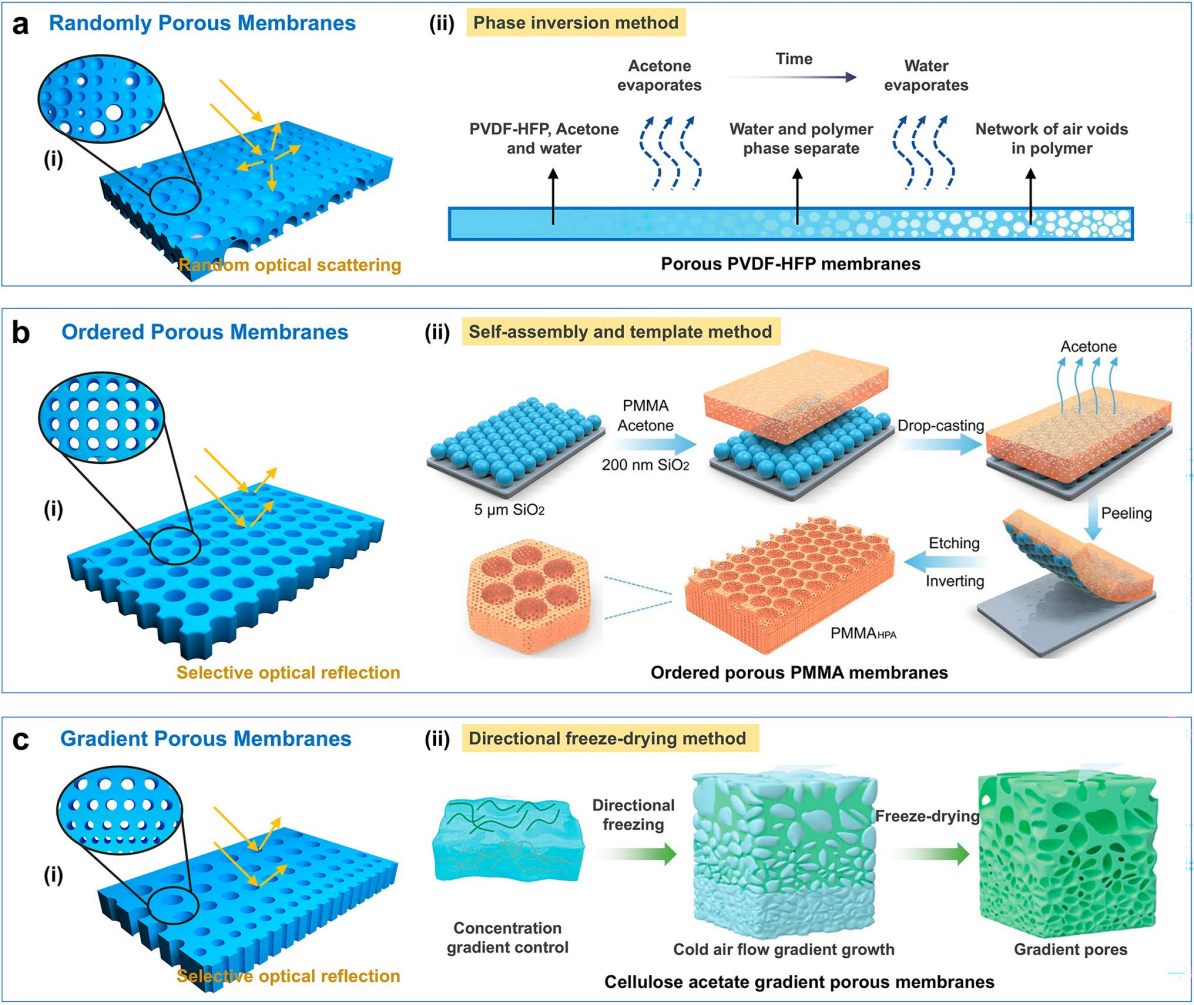
SSDRC porous membranes. **a** (i) Schematic structure of randomly porous membranes. (ii) Schematic diagram for fabricating a PVDF-HFP random porous membrane by a phase-inversion method. Reproduced with permission from [36]. Copyright 2018, AAAS. **b** (i) Schematic structure of ordered porous membranes. (ii) Schematic diagram for fabricating inverse opal PMMA membranes with ordered micropores and random nanopores through self-assembly and template method. Reproduced with permission from [132]. Copyright 2021, Springer Nature. **c** (i) Schematic structure of gradient porous membranes. (ii) Schematic diagram for preparing cellulose acetate gradient porous membranes using directional freeze-drying method. Reproduced with permission from [136]. Copyright 2024, John Wiley and Sons

The structure of a random porous membrane is characterized by uniformly sized pores that are randomly distributed throughout its interior (Fig. 7a-i) [116]. Solar reflectance can be further optimized by tailoring the porous structures to regulate the membrane’s light-scattering properties across the solar spectrum. The pore sizes of the random porous membranes vary from 50 nm to 5 μm, enabling effective scattering of light across the entire solar spectrum. Phase separation is one of the simplest and most effective methods for preparing random porous membranes. Under specific conditions, such as temperature changes, solvent evaporation, or the addition of a non-solvent, a polymer solution undergoes phase separation, forming polymer-rich and solvent-rich phases. These phases eventually solidify into a porous structure. Additionally, the formation and growth of the polymer-rich phase during phase separation are random, leading to a random distribution and size of the pores. For instance, Yang et al. prepared a randomly porous PVDF-HFP membrane through a phase-inversion method (Fig. 7a-ii) [36]. The precursor solution, consisting of PVDF-HFP, acetone (solvent), and water (non-solvent), was coated onto the substrate and allowed to dry naturally in the air. Rapid evaporation of the volatile acetone led to phase separation of PVDF-HFP from water, resulting in the formation of a random porous PVDF-HFP membrane. The resulting PVDF-HFP porous membrane exhibited a high solar reflectivity of 96% and ATW emissivity of 97%, with an average cooling power of ~ 96 W m^−2^ under solar intensity 750 W m^−2^, respectively. In addition, bioinspired materials that mimic biological structures for thermoregulation have shown promise for radiative cooling [117–122]. Many natural organisms [121], including silver ants [123], cocoons [117], golden longicorn beetles [124], lotus leaves [125], white beetles [126], and cicadas [127], exhibit remarkable photonic structures for thermal regulation. For instance, cicada, a thermophilic insect, has been observed to utilize brilliant golden microspikes with a nanophotonic porous heart-shaped structure for radiative cooling in summer [127]. Inspired by this efficient biological prototype, Fan et al. reported a bioinspired porous membrane based on porous thermoplastic polyurethane embedded with alumina (TPU/Al_2_O_3_) nanoparticles. The TPU/Al_2_O_3_ porous membranes featured microscale pores and surface humps, emulating the key characteristics of the cicada microspikes. Moreover, TPU, which had numerous extinction peaks in the infrared ranges, including C-O (1732 cm^−1^), C-N (1533, 1311, and 1223 cm^−1^), and C–O–C (1174 and 1074 cm^−1^), was utilized to enhance the MIR emission. The intentional embedding of Al_2_O_3_ further increased extinction efficiency. The TPU/Al_2_O_3_ membranes demonstrated a high solar reflectivity of 97.6% and an average ATW emissivity of 95.5%. The TPU/Al_2_O_3_ porous membranes exhibited a cooling power of 78 W m^−2^ and achieved a maximum sub-ambient cooling of 6.6 °C at noon. Inspired by the hair structure of longicorn beetles, Zhu et al. developed a photonic film composed of periodically arranged micro-pyramidal polydimethylsiloxane (PDMS) embedded with randomly distributed aluminum oxide ceramic particles [124]. This unique pyramidal architecture significantly enhances solar reflectivity up to 95%, achieving a cooling power of 90.8 W m^−2^. This research offers valuable bioinspired insights into the scalable production of bionic photonic cooling materials.

An ordered porous membrane features a periodic arrangement of pores, resembling a photonic crystal (Fig. 7b-i). The periodic structures can selectively reflect light within a specific wavelength range through Bragg diffraction. Despite their inefficiency in reflecting sunlight across a broad spectrum, they can effectively regulate absorption within the ATW range. By collaboratively designing ordered micropores and disordered nanopores structures, it is possible to achieve both high reflections of sunlight and high emissions in the ATW range. The ordered micropores and disordered nanopores structures can be fabricated by template method, photolithography, and 3D printing [12–131]. Wu et al. prepared an inverse opal PMMA (IO-PMMA) membrane with ordered micropores and random nanopores through a sacrificial template method (Fig. 7b-ii) [132]. PMMA exhibits multiple extinction peaks within the ATW waveband, endowing IO-PMMA membranes with spectrum-selective emission. The periodic reentrant structures and hierarchical nano-/microscale pores helped improve scattering efficiency and increased infrared emission by allowing multiple diffuse reflections at different angles. With these combined attributes, IO-PMMA membranes achieved a high solar reflectivity of 95% and ATW emissivity of 98%, enabling sub-ambient cooling of 5.5 °C under a solar irradiation of 930 W m^−2^. The periodic arrangement of the hierarchical nano/microscale pores can maximize both the surface area and the number of scatters per unit and increase the overall scattering efficiency.

Gradient porous membranes exhibit a gradual change in pore diameter, and porosity along the thickness direction (Fig. 7c-i) [133–135]. The pore size of gradient porous membranes ranges from nanometer to micrometer. When incident light encounters the interface between air and the porous membranes, the gradient in micro-/nanopore sizes selectively interacts with the solar spectrum, including ultraviolet, visible, and near-infrared wavelengths. Hence, a gradient porous structure allows for precise control over the wavelength and direction of sunlight reflection. Mao et al. prepared a gradient structure porous metamaterials (GSPMs) using a cellulose acetate matrix through a step-by-step freeze-casting technique (Fig. 7c-ii) [136]. The arrangement of a gradient porous structure minimized the reflection of infrared photons, thereby enhancing the MIR emission due to the gradual change in refractive index. Three porous metamaterials, downward-GSPM (D-GSPM), upward-GSPM (U-GSPM), and random porous material (RPM) exhibited different ATW emissivity values of 96.9%, 97.6%, and 90.3%, respectively. The electric field distribution was simulated to compare the MIR absorption/emission capabilities of these three porous metamaterials at a representative wavelength of 10 μm. The spectrograms of the absorption field revealed a stronger electric field absorption intensity for the GSPMs compared to the RPM, suggesting that the gradient porous structures enhance the absorption capacity for incident light, thereby improving the MIR thermal emission.

### Particle Coatings

**Fig. 8:**
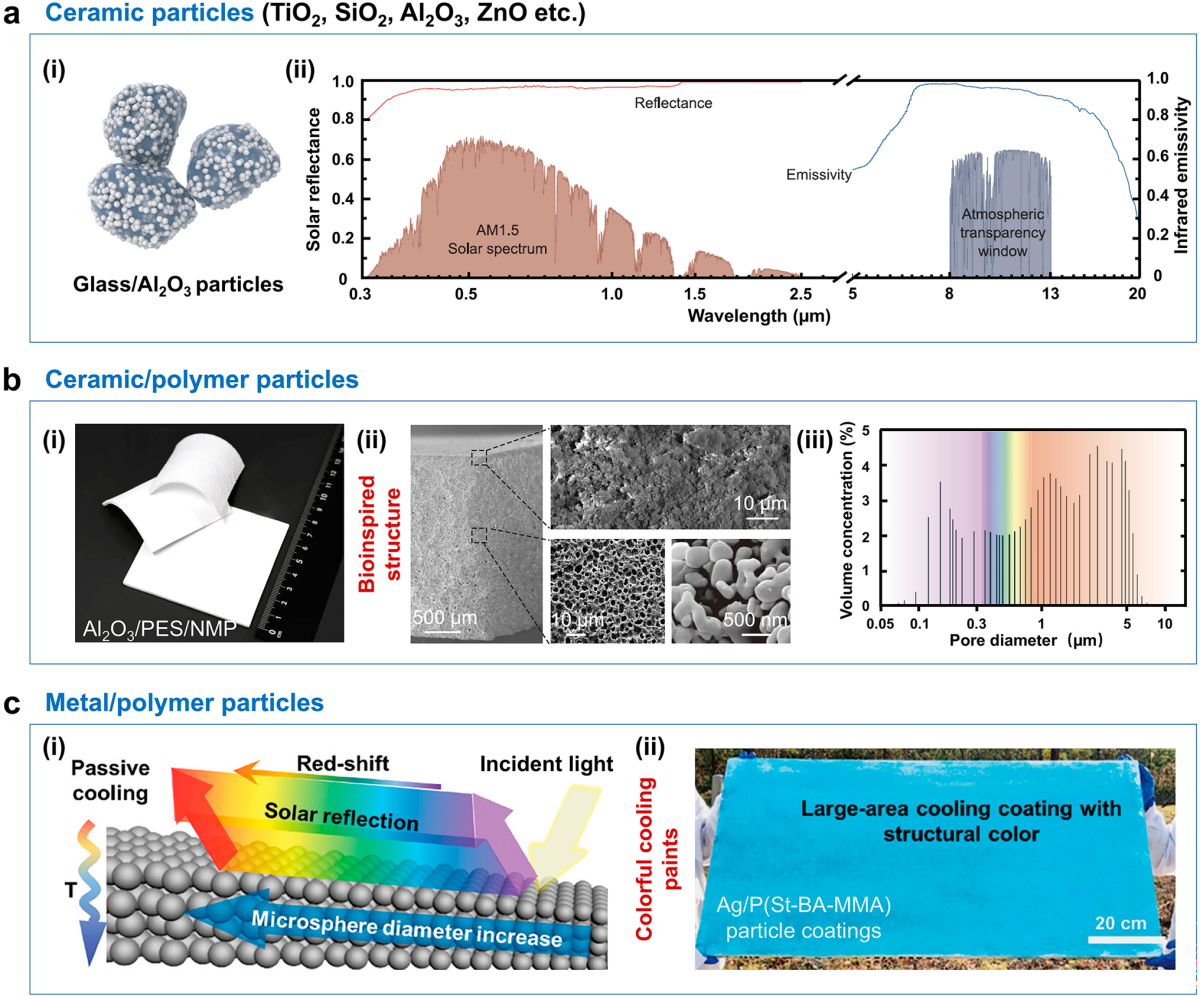
SSDRC particle coatings. **a** Ceramic particles. (i) Schematic diagram of the glass/Al_2_O_3_ cooling particles. (ii) Reflectance/emissivity spectra of glass/Al_2_O_3_ particles in the solar and MIR ranges. Reproduced with permission from [43]. Copyright 2023, AAAS. **b** Ceramic/polymer hybrid particles. (i) Photograph and (ii) SEM images of a polymer/Al_2_O_3_ cooling ceramic particles. (iii) Volume concentration of pores within the cooling ceramics. Reproduced with permission from [44]. Copyright 2023, AAAS. **c** Metal/polymer particles. (i) Schematic diagram of Ag-coated colloidal crystals for building cooling. (ii) Demonstration of large-scale preparation of Ag-coated colloidal crystals. Reproduced with permission from [148]. Copyright 2024, John Wiley and Sons

The solar reflectivity of particle coatings can be effectively regulated by optimizing the size and arrangement of particles, a principle that has been widely explored in the development of high-performance radiative cooling materials. Recent advancements have demonstrated the potential of various particle systems, including ceramic particles [12, 43, 137–140], ceramic/polymer particles [141, 142], and metal/polymer particles [143]. Among these, ceramic particles such as TiO_2_ [137], SiO_2_ [12], Al_2_O_3_ [43], and ZnO [144] stand out due to their exceptional chemical stability and rich infrared-active vibrational modes (e.g., Si–O, Al–O, Zn–O). These vibrational modes, which occur within the atmospheric transmission window, enable spectrally selective radiative cooling, making ceramic particles a promising candidate for such applications. Al_2_O_3_ particles, in particular, exhibit remarkable properties, including a high melting point (2072 °C), refractive index (1.7), and bandgap (7.2 eV), coupled with excellent thermodynamic and chemical stability. These attributes make them ideal for use as anti-sintering agents in cooling glass materials. For instance, Hu et al. developed a cooling glass through a straightforward two-step method [43]. In this process, inexpensive glass and Al_2_O_3_ particles were mixed to prepare a slurry, which was then subjected to thermal annealing of glass to produce cooling coatings (Fig. 8a). This approach leveraged the infrared-active vibrational modes of glass particles within the ATW region, which acted as unconventional binders, forming a robust porous framework (~ 12 mm in size) that enhanced selective ATW emission through phonon-polariton resonance. The inclusion of Al_2_O_3_ particles (mean size of 0.5 mm) further improved solar reflectance via Mie scattering. The resulting glass/Al_2_O_3_ composite coatings achieved a solar reflectance of > 96% and an ATW emissivity of ~ 95%, with no degradation in cooling performance even after 60 days of water immersion. This highlights the material’s exceptional moisture stability and potential for long-term outdoor applications. While micro- and nanoscale ceramic materials are widely used in SSDRC due to their durability, high hardness, and selective emittance, their inherent lack of adhesion and low mechanical strength pose significant challenges for outdoor use. To address these limitations, researchers have increasingly focused on hybrid systems that combine ceramic particles with polymer matrices. These composites not only enhance cooling efficiency but also improve mechanical robustness and adhesion [145–147]. For example, Tso et al. developed a radiative cooling ceramic, composed of PES, N-methyl-2-pyrrolidone (NMP), and Al_2_O_3_ (Fig. 8b) [44]. The cooling ceramics featured a distinctive densely packed outer layer and numerous internal voids. They were prepared through a combination of phase inversion and sintering processes. During the phase inversion, a polymer-rich membrane was created, forming an anisotropic porous network. Subsequent high-temperature sintering facilitates the bonding of Al_2_O_3_ particles, resulting in a precisely preserved porous structure. The resulting porous Al_2_O_3_ network exhibited a near-perfect solar reflectivity of 99.6% and a high ATW emissivity of 97%, attributed to the vibrational modes of Al-O chemical bonds (9.5–12.1 µm). This material demonstrated consistent sub-ambient cooling outdoors, with a cooling power exceeding 130 W m^−2^ at noon, showcasing its potential for practical applications. In addition to ceramic-based materials, metallic components have also been explored for their high solar reflectance. For instance, Chen et al. developed silver-coated colloidal photonic crystal (Ag/CPC) metamaterial coatings, which exhibited highly selective reflection of solar radiation (Fig. 8c) [148]. The Ag/CPC coatings achieved an average solar reflectivity of 73% and an ATW emissivity of 91%, with a theoretical cooling power of 30.4 W m^−2^. Notably, these coatings could be applied to architectural walls, offering a vibrant blue color that expands their potential use in aesthetically appealing radiative cooling paints.

Multilayer structures have been engineered into radiative coolers via periodic stacks of particle-coating layers [53, 149]. A multilayer radiative cooler typically comprises vertically stacked particle coatings designed to suppress solar absorption while enhancing infrared emission. This architecture functions as a 1D photonic crystal, where alternating layers of materials with distinct refractive indices and thicknesses are arranged periodically [86]. The multilayer strategy exploits photonic bandgap effects to precisely tailor electromagnetic responses. By optimizing material combinations and layer thicknesses, these structures achieve targeted reflection spectra with high solar reflectivity. For instance, Fan et al. demonstrated a multilayer photonic reflector exhibiting 97% solar reflectivity, comprising seven alternating HfO_2_/SiO_2_ layers with sub-100 nm thicknesses deposited on a 200 nm silver-coated silicon substrate [7]. In this design, HfO_2_ acts as a high-refractive-index layer with minimal ultraviolet absorption, whereas SiO_2_ serves as a low-index, optically transparent layer. Moreover, SiO_2_ and HfO_2_ exhibited selective emission in the 8–13 µm wavelength range. The HfO_2_/SiO_2_ photonic reflector demonstrated high solar reflectance and spectrum-selective emission. The dimensional optimization of such multilayer systems involves intricate adjustments of layer count, material selection, and individual thicknesses. Machine learning has emerged as a powerful tool for this purpose. Rho et al. employed genetic algorithms to optimize multilayer emitters for daytime passive radiative cooling [53]. The optimization approach is expected to increase additional degrees of freedom. Among the four material candidates of SiO_2_, silicon nitride (Si_3_N_4_), magnesium fluoride (MgF_2_) and HfO_2_, proper materials were recommended, and thicknesses were optimized for desired optical functionalities. Machine learning algorithms have also demonstrated usefulness in the design of multilayer radiative cooling materials. Table 3 summarizes some of the reported SSDRC materials, detailing their preparation techniques, optical characteristics, and cooling performances. Collectively, these advancements highlight the importance of material selection and structural design in optimizing radiative cooling performance. However, challenges such as durability, aesthetics, and tunability of SSDRC materials remain critical areas for future research. By addressing these issues, the field can move closer to the widespread adoption of radiative cooling technologies in real-world applications.

## Applications of SSDRC

The multifunctionality of SSDRC materials significantly expands their application scenarios. In this section, we examine the recent advancements in SSDRC materials, with a specific focus on their applications in personal thermal management, outdoor building cooling and energy harvesting.

### Personal Thermal Management

**Fig. 9:**
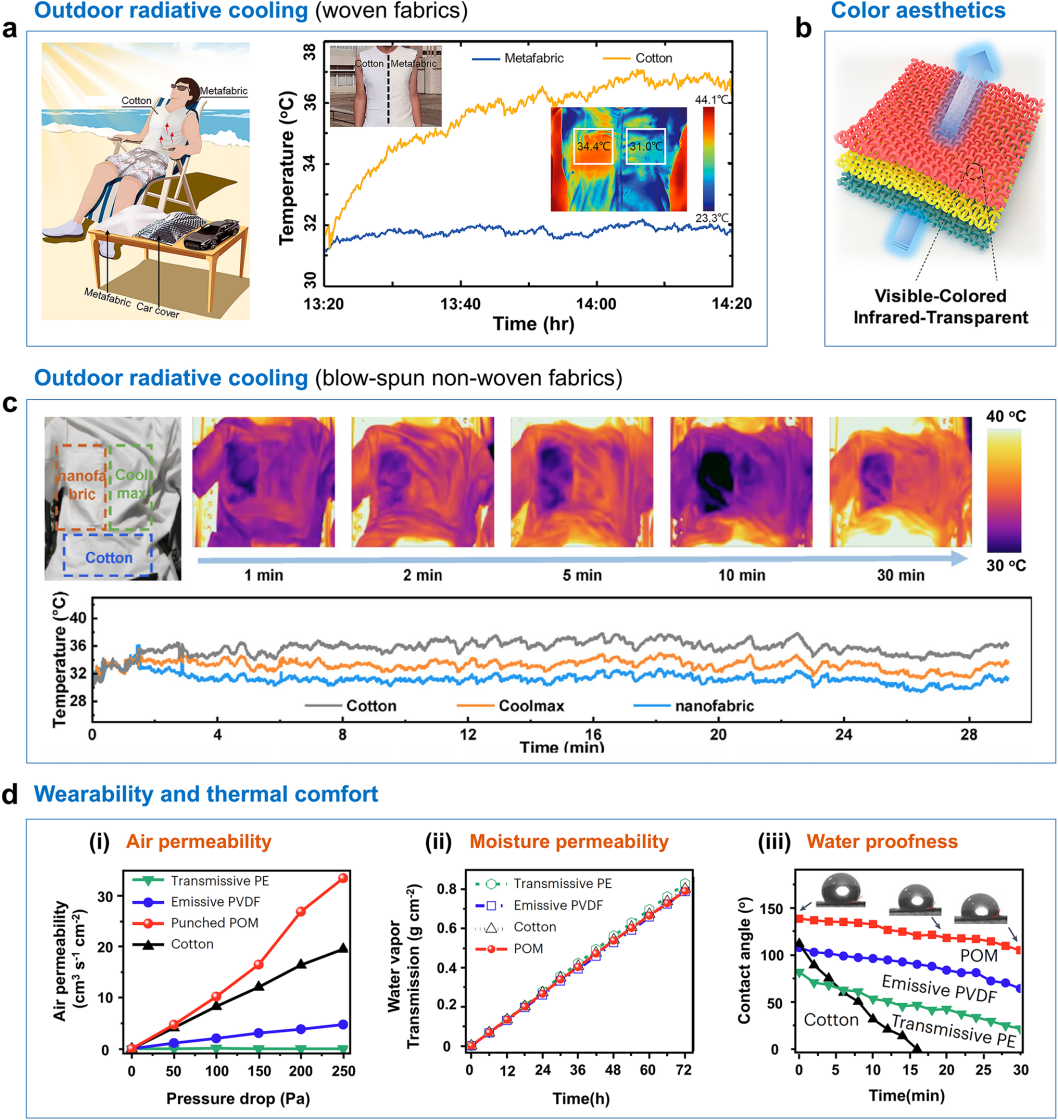
Personal thermal management by radiative cooling. **a** Woven fabrics that exhibit daytime radiative cooling under solar irradiation at noon. Reproduced with permission from [98]. Copyright 2021, AAAS. **b** Fabrication of colored radiative cooling textiles through inorganic pigment. Reproduced with permission from [166]. Copyright 2019, Elsevier. **c** Large-area non-woven fabrics that exhibit daytime radiative cooling under direct solar irradiation at noon. Reproduced with permission from [93]. Copyright 2024, Elsevier. **d** Comparison of the wearability of the polyformaldehyde nanofibers, commercial cotton, emission-type PVDF membranes, and transmission-type PE textiles, including air permeability, water vapor transmission, and water contact angle tests. Reproduced with permission from [41]. Copyright 2023, Springer Nature

In recent years, we have seen advancements in personal thermal management (PTM), focusing on regulating heat transfer in our immediate surroundings, clothing, and skin [149–157]. SSRDC materials have been incorporated into cooling textiles for PTM applications. Key considerations for SSRDC fabrics in PTM include cooling performance during hot midday conditions, wearing comfort, and color aesthetics [158, 159]. Tao et al. reported a multilayer metamaterial fabric [98]. As shown in Fig. 9a, a volunteer wearing a metamaterial fabric vest reclined under direct sunlight for an hour, and the thermal properties of the vest and the volunteer were monitored in real time. The use of metamaterial fabric vests resulted in a noticeable reduction in body temperature by approximately 4 °C compared to wearing a common cotton vest. During the half-hour test, the surface of the metamaterial fabric vest gradually exhibited a notable temperature difference, demonstrating long-term cooling stability. Furthermore, the tunability of visible color is another critical factor that enhances the marketability of SSDRC textiles [159–165]. Cui et al. reported a strategy that employed inorganic nanoparticles as a coloring component for producing brightly colored, infrared-transparent textiles (Fig. 9b) [166]. The as-fabricated textiles not only showed a high infrared transparency of ~ 80% and a passive cooling effect of 1.6–1.8 °C but also exhibited intense visible colors with good washing stability. However, these infrared-transparent colored textiles demonstrated efficacy in indoor passive cooling but were not suited for outdoor radiative cooling applications. Rho et al. introduced a colored daytime radiative cooler that achieved high near-infrared reflectance and high atmospheric window emissivity, while enabling the generation of subtractive primary colors through Fabry–Pérot interference in metal–insulator-metal structures. This innovative design offers a promising solution for applications requiring both effective radiative cooling and aesthetic color customization [167]. Our group fabricated radiative cooling nanofabrics comprising SiO_2_ nanofiber films and expanded polytetrafluoroethylene films (Fig. 9c) [93]. The nanofabrics featured hierarchical structures, exhibiting high solar reflectivity (94%) and high ATW emissivity (94%). To assess the practical cooling effectiveness of nanofabrics, a custom-made vest incorporating commercial cooling fabrics (Coolmax fabrics, cotton fabrics) and nanofabrics was crafted. A volunteer wore the vest in direct sunlight for 30 min, and an IR imager monitored the temperature distribution of the vest. The temperature curves measured for nanofabrics, cotton fabrics, and Coolmax indicate the surface temperature of all three samples stabilized after 5 min. The results illustrated that nanofabrics have the capability of inducing a cooling sensation. Therefore, a feasible method is to combine infrared-transparent colored textiles [168] with radiative cooling nanofabrics, thereby achieving synergy between outdoor radiative cooling and color aesthetics. Moreover, the wearability performance of SSDRC fabrics should be taken into consideration for practical applications. Figure 9d shows the permeability and antifouling performances of polyoxymethylene (POM) nanofibers [41]. The air permeability of POM nanofibers reached 34 cm^3^ s^−1^ cm^−2^, outperforming PE and PVDF membranes. In addition, the POM nanofibers showed high water vapor transmission rate (WVTR) (0.011 g cm^−2^ h^−1^), which was similar to commercial cotton (~ 0.012 g cm^−2^ h^−1^). The anti-humidity properties, crucial for maintaining textile dryness and cleanliness in humid conditions, were also evaluated. The water contact angle of POM nanofibers was measured at 138° and remained at 122° after 30 min, significantly higher than other textiles, indicating their superior waterproofing and anti-humidity performance. However, the hygroscopicity and moisture breathability of non-woven materials remain challenging compared to traditional fabrics, making them unsuitable for long-term wear. These issues can be solved by incorporating softeners to enhance the flexibility of non-woven fabrics and improving their hygroscopicity through chemical modifications.

The urban heat island effect has become a significant environmental challenge, particularly in its impact on human thermoregulation and its synergistic interaction with global warming. This phenomenon is primarily driven by the differential thermal properties between urban structures and natural landscapes [169]. Specifically, anthropogenic surfaces, including buildings and paved areas, exhibit higher solar radiation absorption coefficients and thermal emissivity compared to vegetative cover and aquatic systems. This results in elevated surface temperatures that frequently exceed human skin temperature, creating a thermal gradient that promotes heat transfer to the human body. Broadband emissive textiles often experience performance degradation due to parasitic thermal radiation from surrounding urban elements. In contrast, SSRDC textiles have demonstrated exceptional capability in mitigating these thermal loads by effectively suppressing non-ATW parasitic heat from the surrounding ground or buildings [25]. The evolution of SSRDC technology is progressing toward adaptive thermal management systems, incorporating dynamic thermal regulation mechanisms and wearable electronic integration. Dynamic thermal-regulating textiles can utilize various modes of thermal regulation to adapt to changes in environmental temperature. For instance, these textiles can autonomously cool the human body during the summer and provide warmth in the winter. Thermochromic and electrochromic materials have been used in smart dynamic thermal regulation [169–172]. Additionally, SSDRC textiles can be integrated with flexible electronics, including wearable batteries, moisture sensors, and temperature sensors, to enhance comprehensive healthcare applications [49, 150, 172–176]. Future efforts are expected to integrate various desired functions, such as smart thermal management, self-powering, sensing, and computing into clothing.

### Outdoor Building Cooling

**Fig. 10:**
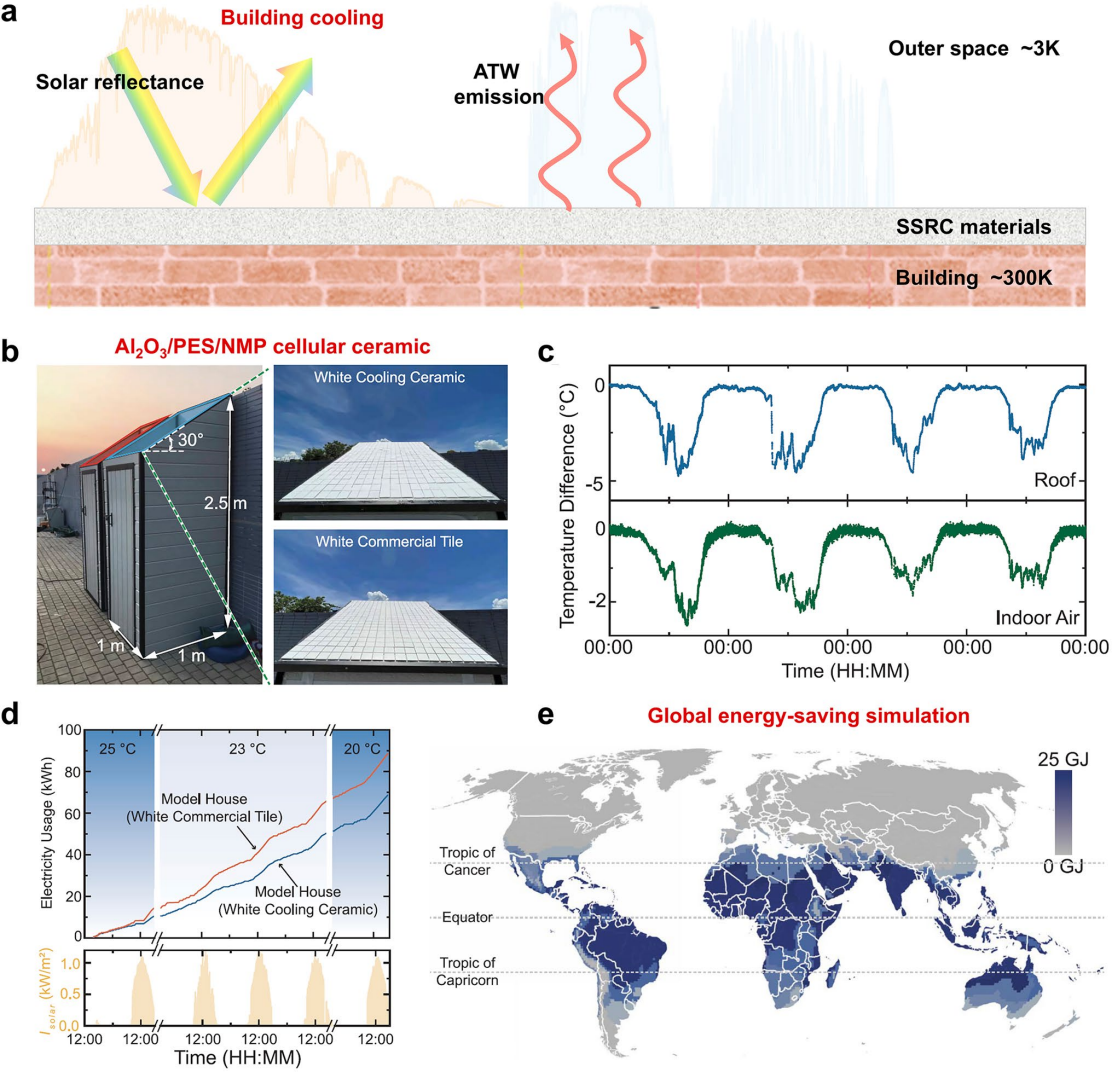
Outdoor building cooling. **a** Schematic of the building cooling of SSDRC paints through solar reflection and selective MIR emission. **b** Photographs of two model houses, with the white cooling ceramic and white commercial tile applied on the roof (area: ~ 1.15 m^2^). **c** Differences in the roof and indoor air temperatures for the two model houses. **d** Electricity usage of the two houses with air-conditioning set points of 25 °C, 23 °C, and 20 °C. **e** Energy-saving performance on a worldwide scale considering the energy consumed by cooling systems, fans, and heating equipment. Reproduced with permission from [44]. Copyright 2023, AAAS

As global warming and escalating energy consumption contribute to rising global temperatures, the need to cool living environments has become increasingly urgent. In this context, SSDRC ceramic particles, such as Al_2_O_3_, SiO_2_, TiO_2_, and their composites, have emerged as promising candidates for the development of radiative cooling paints. These materials hold significant potential for large-scale application on buildings, offering a pathway to reduce electricity consumption for cooling and mitigate the urban heat island effect (Fig. 10a) [44, 176–180]. Tso et al. reported cellular ceramic materials composed of Al_2_O_3_/PES/NMP particle coatings [44]. Their study not only demonstrated the material’s cooling performance but also explored its practical application in real-world building scenarios. For example, two identical model houses were constructed, one roofed with Al_2_O_3_/PES/NMP ceramic and the other with commercial tiles (Fig. 10b). Over a four-day test, the ceramic-coated roof maintained a temperature 5 °C lower than the tiled roof, highlighting its effectiveness in reducing heat absorption (Fig. 10c). This finding underscores the potential of SSDRC ceramics to significantly improve thermal comfort in buildings, particularly in regions with high cooling demands. To quantify the energy-saving potential of the engineered cooling ceramic, researchers conducted additional assessments by operating air-conditioning units in the model houses in the summer. The air conditioning electricity usage over three periods, with set temperatures of 25, 23, and 20 °C was monitored in real time (Fig. 10d). These findings not only validate the material’s cooling efficiency but also suggest its potential to contribute to global energy conservation efforts. The Al_2_O_3_/PES/NMP ceramic model house consumed less electricity, with energy savings of 26.8%, 22.6%, and 19.6%, respectively. Researchers also simulated energy consumption in full-scale buildings to evaluate the global energy-saving potential of Al_2_O_3_/PES/NMP ceramic walls and roofs. In particular, annual energy savings from indoor air conditioning exceeded 10%, amounting to 25 GJ per year, in the extremely hot regions of South America, North Africa, and South Asia (Fig. 10e). SSDRC architectural coatings can significantly reduce the external surface temperatures of buildings, thereby reducing substantial electricity consumption generated by air conditioning. Additionally, radiative cooling particles often suffer from material mismatch when applied to building surfaces, especially on cement-based materials like concrete [181]. This mismatch can cause interfacial detachment, limiting their practical use in passive building cooling. To address this, Cui et al. proposed a particle-solid transition architecture [182]. They achieved this by welding BaSO_4_ nanoparticles onto a cement-based solid substrate. In this design, BaSO_4_ nanoparticles are partially exposed, enabling radiative cooling, rather than being fully embedded in the matrix. This approach avoids the screening effect typically caused by cementitious substrates in uniform composites. At the same time, the solid substrate shares material properties with cement-based building surfaces, eliminating the interface mismatch common in traditional radiative cooling coatings, membranes, or bulk materials. This design combines effective radiative cooling with enhanced compatibility for building applications. In addition, the transformation of waste plastics into building cooling materials with radiative cooling properties has also garnered significant attention. Zhang et al. [183] proposed quickly converting abundant waste polystyrene foam into cooling coating via a closed-loop solvent extraction strategy to satisfy the scale and clean production requirements of building a cooling envelope. Results demonstrate the coating has a randomly porous structure thus reflecting 97% solar radiation and emitting 94% thermal radiation. About 8 °C of net and 7.5 °C/1000 cm^3^ building space cooling capacity can be achieved even under 1500 W m^−2^ of solar radiation. This work will effectively inspire the development of large-scale and low-environmental-impact building cooling envelopes. Recently, there have still many challenges in practical applications, one of which is the durability issue of SSDRC architectural cooling coatings. These coatings experience prolonged exposure to outdoor conditions, such as rain, acidic and alkaline environments, and ultraviolet radiation, all of which can considerably impact their stability. Secondly, in practical applications, SSDRC architectural cooling coatings are expected to provide cooling/warming selectivity, effectively radiating heat in hot weather and offering insulation in cold weather. In the quest for sustainable architecture, smart building coatings that can dynamically regulate cooling and warming have emerged as highly desired innovations.

### Energy Harvesting

**Fig. 11:**
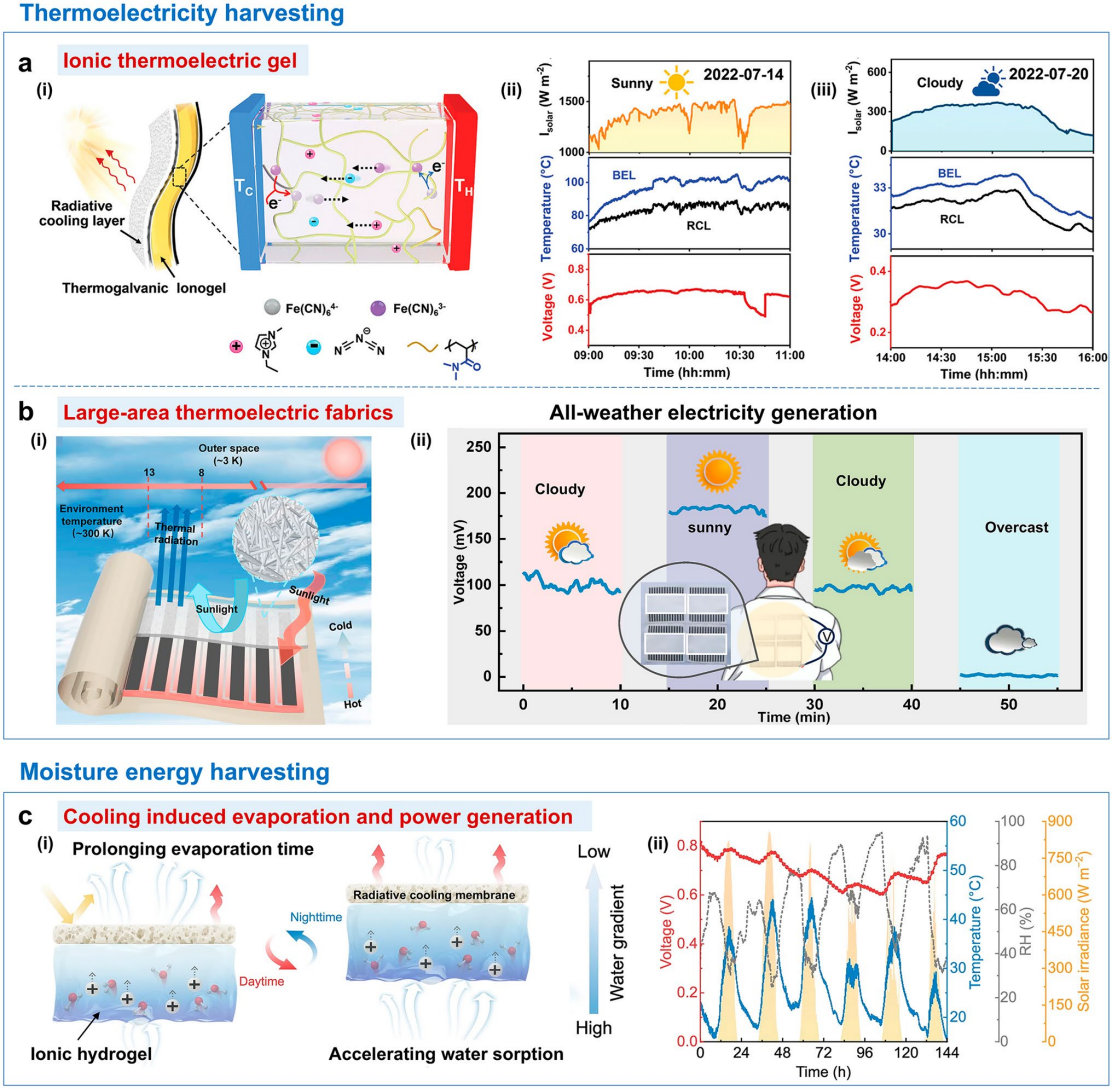
Radiative cooling enhanced energy harvesting. Thermoelectric energy harvesting. **a** Ionic thermoelectric gel. (i) Schematic of enhanced thermoelectricity by daytime radiative cooling. Detailed solar intensity and temperature data of the two sides of the thermogalvanic ionogel and the voltage change on (ii) sunny and (iii) cloudy days. Reproduced with permission from [45]. Copyright 2024, John Wiley and Sons. **b** Large-area radiation-modulated thermoelectric fabrics. (i) Principle and the structures of radiation-modulated thermoelectric fabrics. (ii) Output voltage generation performance of self-powered wearable textile under four different weather conditions. Inset, schematic diagram of applications in self-powered wearable textile. Reproduced with permission from [52]. Copyright 2025, AAAS. Moisture energy harvesting. **c** Radiative cooling induced cycle evaporation and power generation. (i) Schematic diagram illustrating the structure of the laminated ionic hydrogel. The operational mode of the laminated ionic hydrogel in the diurnal cycle. (ii) Continuous moisture energy-harvesting test results for 6 days outdoors. Reproduced with permission from [191]. Copyright 2024, Springer Nature

Recent advancements in radiative cooling have demonstrated its potential for integration with various sustainable energy-harvesting technologies, such as thermoelectric harvesting [174, 183–189], moisture-electricity harvesting [51] and triboelectric energy harvesting [190]. These integrations not only enhance the functionality of radiative cooling materials but also open new avenues for energy generation and sustainability. For instance, Yan et al. developed a thermocell device by integrating thermogalvanic ionogel (THG-ionogel) with DRC materials [45]. The device consisted of a radiative cooling layer (DRCL), a THG-ionogel, and a black encapsulation layer (BEL) that functioned as a radiative heating layer (Fig. 11a). The generation of thermoelectricity under diverse weather conditions was investigated through real-time testing conducted outdoors. As shown in Fig. 11a-ii, on a sunny day, the DRCL of the thermocell demonstrated an average sub-ambient, ΔT of 8.3 °C, generating an average voltage of 0.61 V. While on a cloudy day (Fig. 11a-iii), with fog and haze limiting radiative heat transfer into the atmosphere, the average ΔT decreased to 1.5 °C and the corresponding output voltage reached 0.28 V. These results highlight the adaptability of thermocell devices to varying environmental conditions. The integration of radiative cooling with flexible thermoelectric systems represents another promising direction, particularly for applications in wearable technology. Hence, our group present a facile approach involving the screen printing of large-scale carbon nanotube (CNT)-based thermoelectric arrays on conventional textile. The operating mechanism of radiation-modulated thermoelectric textiles is illustrated in Fig. 11b-i. CNT arrays acted as both photothermal and thermoelectric devices, while PVDF-HFP membranes served as radiative cooler that reflect sunlight and dissipate heat. The controlled coverage ratio of the PVDF-HFP SSDRC membranes on the CNT-based thermoelectric arrays allows precise adjustment of the temperature gradient across the thermoelectric array, enhancing energy convention performance from thermal to electrical energy. Four pieces of radiation-modulated thermoelectric fabrics were assembled on clothing to explore its thermoelectric performance. Typically, the output voltage could reach 115.2, 184.5, 100.6, and 2.9 mV at four different times on a clear day, respectively (Fig. 11b-ii). In addition, a strategy of combining radiative cooling with moisture energy harvesting was reported by Zhao’s group [191]. As shown in Fig. 11c, the moisture energy-harvesting device was characterized by a bilayer polymer, composed of a hydrophobic porous PVDF-HFP layer and a hygroscopic ionic hydrogel (IH) layer (PP/IH). The PP/IH laminated hydrogel enabled moisture-electricity generation from the hydrological cycle through the process of water/ion flow, which was driven by thermal exchange with the ambient environment. The asymmetric hygroscopic structure of the PP/IH laminated hydrogel contributed to the establishment of internal gradients in water content and ion concentration, which in turn generated continuous water/ion flow. This continuous flow of ions led to stable electricity generation. During the day, the PVDF-HFP layer blocked the absorption of sunlight, thereby reducing the evaporation of moisture within the PP/IH laminated hydrogel. This prevented the water/ionic concentration gradient within the PP/IH laminated hydrogel from decreasing due to daytime moisture evaporation. At night, the hygroscopic ionic hydrogel absorbs moisture from the environment. The heat generated by the moisture absorption of ionic hydrogel was dissipated into the environment through nighttime radiative cooling of the top PVDF-HFP layer, thereby maintaining a balance of ion concentration gradient. Consequently, the PP/IH laminated hydrogel maintained a stable ion concentration gradient both during the day and at night, thus ensuring a stable voltage output. This dual-day-and-night operation allowed the PP/IH laminated hydrogel to achieve continuous energy output for six days in outdoor experiments. The primary advantage of radiative cooling in thermoelectric energy harvesting is its ability to create a stable temperature difference through radiative cooling, thereby facilitating continuous thermoelectric conversion. In moisture energy harvesting, the incorporation of ionic hydrogel materials enhances energy generation efficiency by preserving water content and ion concentration gradient within the ionic hydrogel.

## Conclusions and Perspective

In recent years, daytime radiative cooling has emerged as a significant area of interest within thermal management research, exhibiting its potential for a wide range of applications, involving personal thermal management, building cooling, and energy harvesting. Daytime radiative cooling materials are designed to achieve high solar reflectivity, minimizing heat absorption from sunlight. They also exhibit strong mid-infrared emissivity, particularly within the ATW region, to efficiently radiate heat into the cooler atmosphere. These materials are often designed to emit broadly across the entire MIR spectrum, but this broad-spectrum emissivity inadvertently leads to unintended absorption of thermal radiation in the non-ATW wavelength band, which can significantly limit the cooling performance. SSDRC materials are tailored to emit primarily within the ATW while suppressing emission/absorption in the non-ATW range, having superior cooling efficiency. In this paper, we review the advancements in SSDRC materials, encompassing their fundamental properties, structural characteristics, fabrication processes, and diverse applications.

**Fig. 12:**
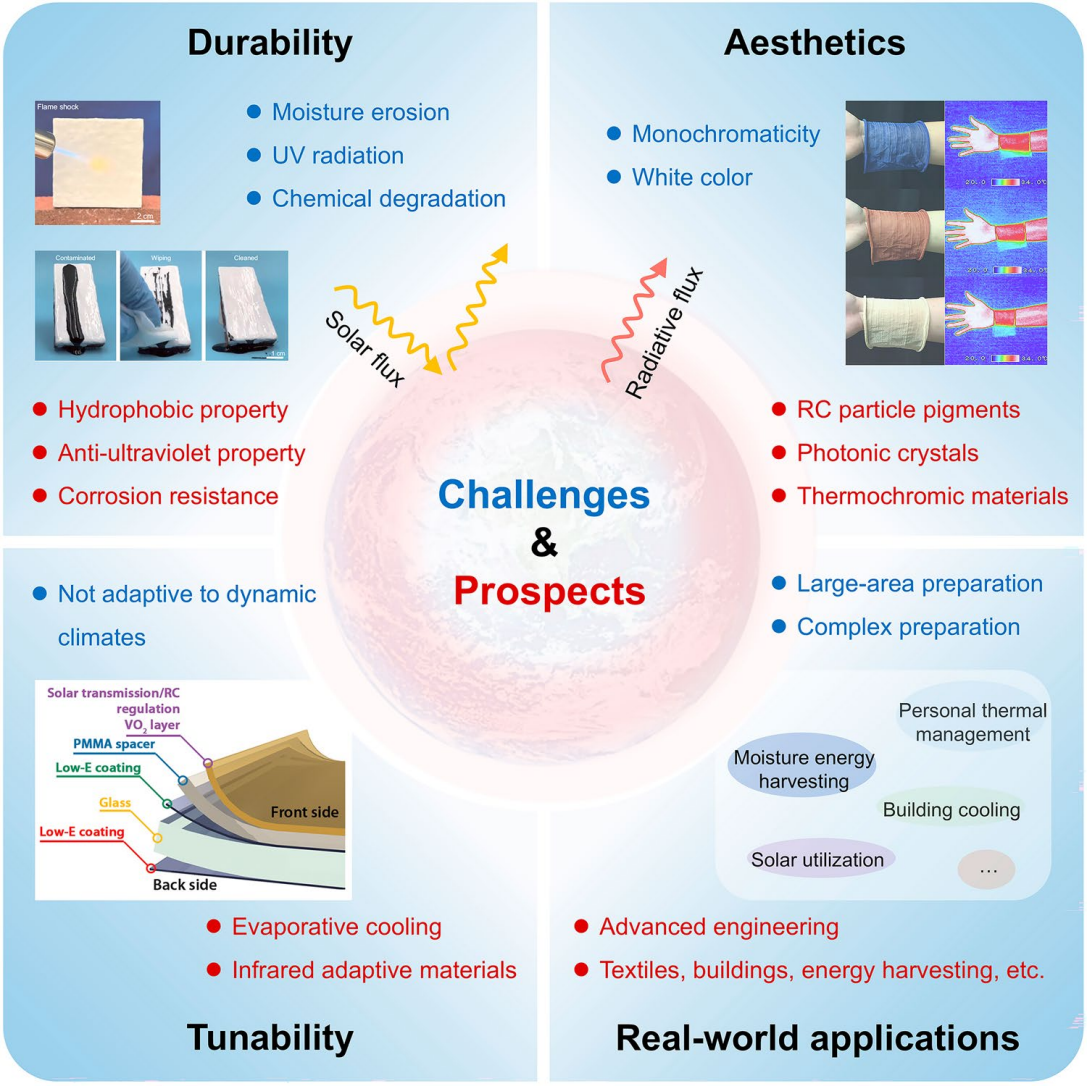
Challenges and prospects of SSDRC materials: exploring high-durability SSDRC materials, fabricating colored SSDRC materials, developing dynamic radiative management, and novel applications

Despite significant achievements in SSDRC materials, challenges that affect their performance and implementation toward practical applications still exist. To facilitate the development of SSDRC materials, we have outlined the general challenges and prospects (Fig. 12). The first challenge is the durability of SSDRC materials, specifically their optical and mechanical properties. Often exposed to outdoor environments, these materials are frequently subjected to various harsh environmental factors, including moisture, ultraviolet, and chemical degradation. To expand their lifespan, it is crucial to enhance their durability through improved hydrophobic properties, ultraviolet reflectivity, mechanical strength, and corrosion resistance. Recent advancements in micro-/nanomanufacturing technologies and interdisciplinary research have yielded promising strategies for durability enhancement [29]: (1) Utilization of environmentally stable ceramic raw materials [100, 108]; (2) Implementation of chemical modification technologies [181, 192, 193]; and (3) Development of hybrid material systems [124]. For example, Wan et al. fabricated superhydrophobic silica fibrous films through electrospinning and calcination [100]. The silica fibrous film not only demonstrated sub-ambient cooling performance of 6 °C, but also exhibited self-cleaning, anti-mildew, and anti-acid abilities. Zhu et al. prepared potassium titanate (K_2_Ti_6_O_13_) doped porous PEO fiber film through electrospinning. This innovative approach significantly improved UV resistance by enabling the K_2_Ti_6_O_13_ nanofibers to absorb high-energy UV photons [194].

Secondly, current traditional SSDRC materials are predominantly white to achieve high solar reflectance. It is challenging to satisfy aesthetic preferences in real-life applications [17]. The color of an SSDRC material, when exposed to sunlight, is typically determined by its absorption of visible-spectrum wavelengths. However, the challenges arise as visible-light absorption tends to heat the SSDRC material, making it difficult to maintain a specific color for aesthetic purposes while preserving effective radiative cooling. A promising solution that relies on diffuse scattering rather than absorption to create a color appearance while maintaining high sunlight reflectance, has been proposed. For example, Wang et al. proposed a cooling structure design consisting of a TiO_2_/SiO_2_ multilayer, a frosted glass disordered layer, and a reflective silver mirror [195]. The TiO_2_/SiO_2_ multilayer efficiently reflects undesired light while allowing high transmittance for the desired light. For example, a blue sample transmits blue light while strongly reflecting other visible lights. The frosted glass and silver mirror diffusely reflect the transmitted light, which then passes back through the multilayer, leading to a highly saturated color appearance. This structural design maximizes daytime radiative cooling performance while achieving specific visible-light colors. Based on structural classification, colored SSDRC materials can be categorized into two primary types: photonic crystal-based and pigment nanoparticle-based systems [196]. Photonic crystals, as a class of optical metamaterials, offer a versatile platform for precisely engineering spectral properties in radiative cooling applications. Representative examples include Ag-SiO_2_-TiO_2_ [160], Ag-SiO_2_-Si_3_N_4_ [197], and Si-SiO_2_-TiO_2_ [198] multilayer photonic crystals, which have been successfully implemented in colored daytime radiative cooling. In contrast, nanoparticle-based structures demonstrate superior processability, where pigment nanoparticles act as randomly distributed optical resonators when embedded in polymer matrices. Commonly employed nanoparticles, such as SiO_2_ [199], TiO_2_ [200], ZnO, and BaSO_4_, have been effectively integrated to fabricate colored SSDRC materials.

Moreover, fluctuating weather conditions are another factor that restricts SSDRC applications, as their cooling power is highly susceptible to weather conditions. Combining SSDRC with other technologies such as evaporative cooling using hydrogels or hygroscopic salts, serves as a promising strategy for tunability of SSDRC materials. Passive water-based evaporative cooling, which uses water as a refrigerant, is gaining significant attention [201]. This cooling method can be implemented through three main approaches: direct evaporative cooling (DEC) [202], cyclic sorption-driven liquid water evaporative cooling (CSD-LWEC) [203], and atmospheric water harvesting-based evaporative cooling (AWH-EC) [204]. DEC is less favored due to its high consumption of liquid water. In contrast, CSD-LWEC and AWH-EC offer more sustainable alternatives by either recycling the generated vapor or extracting vapor from the ambient air using regenerable water vapor sorbents, such as high-hygroscopicity hydrogels (polyacrylamide [205], sodium polyacrylate [206]) or hygroscopic salts (Ca^2+^ [207], Li^+^ [208], quaternary ammonium salts [203]). These methods enable evaporative cooling without the need for liquid water consumption. The material design of the absorbent material plays a crucial role in the effectiveness of both CSD-LWEC and AWH-EC systems. Chen et al. developed a lithium bromine-enriched polyacrylamide hydrogel for semiconductor cooling [205], in which LiBr worked as the sorbent for atmospheric water capture and polyacrylamide hydrogel provided a solid platform that constrained the solution in a desired region. The hydrogel could reduce the cell phone chip temperature by 15 °C under the heat flux of 2229 W m^−2^. An innovative solution for achieving tunable SSDRC materials lies in the development of infrared adaptive materials. These materials can precisely regulate their infrared optical characteristics in response to seasonal changes and regional climate conditions [209, 210]. For this purpose, various infrared adaptive material systems have been investigated and adapted in radiative cooling, such as graphene [211], electrochromic materials [212], metamaterials [213], ferroelectric materials [214], and phase-change materials (vanadium dioxide [215], liquid crystal materials [216]). For instance, Feng et al. demonstrated a bacterial cellulose-templated radiative cooling liquid crystal membrane [216]. This membrane not only rapidly responds to changes in ambient temperature and adaptively modulates its solar transmittance (from 300 to 2500 nm) but also strongly scatters solar radiation in its opaque state. Owing to the combination of high ATW emissivity of cellulose and adaptive solar transmittance modulation of liquid crystal, this bacterial cellulose-templated radiative cooling liquid crystal membrane holds great potential for thermal regulation applications in smart windows for vehicles and buildings. In contrast to conventional static radiative coolers, infrared adaptive materials exhibit dynamic optical performance, offering the potential for tunable radiative regulation.

Finally, the significant challenge for SSDRC materials lies in achieving scalable manufacturing and widespread implementation while maintaining optimal performance characteristics. This critical hurdle encompasses not only the technical aspects of large-scale production but also the practical considerations of cost-effectiveness, material availability, and integration with existing infrastructure systems. The successful transition from laboratory-scale prototypes to commercially viable, mass-producible solutions represents a pivotal step in realizing the full potential of SSDRC technologies for global applications. Tao et al. developed large-scale woven metafabrics [98]. These metafabrics are cost-effective and easy to produce, making them suitable for smart textiles and passive radiative cooling. Their compatibility with existing textile manufacturing processes supports widespread use. SSDRC materials can be widely applied in urban infrastructure. A representative example is the white coating created by Hu’s team [43]. They mixed micrometer-sized Al_2_O_3_ particles with glass particles in a solvent. This coating can be painted onto different surfaces using simple brushes, enabling large-scale applications. It works for both personal cooling and building cooling, meeting energy-saving needs in daily life and cities. The development of SSDRC materials represents a transformative step toward addressing global cooling challenges. Innovations such as the metafabrics for wearable applications and the Al_2_O_3_-based coating for architectural cooling demonstrate the potential of these materials to revolutionize both personal and urban thermal management. As research progresses, the focus on scalability, cost-effectiveness, and ease of application will be critical to ensuring the widespread adoption of these technologies. By leveraging these advancements, SSDRC materials can play a pivotal role in creating a more sustainable and energy-efficient future, ultimately contributing to a cleaner and cooler planet.
